# MCM6 is a critical transcriptional target of YAP to promote gastric tumorigenesis and serves as a therapeutic target

**DOI:** 10.7150/thno.75431

**Published:** 2022-09-06

**Authors:** Yifei Wang, Huarong Chen, Weixin Liu, Huan Yan, Yihan Zhang, Alvin H.K. Cheung, Jinglin Zhang, Bonan Chen, Li Liang, Zhaocai Zhou, Chi Chun Wong, William K.K. Wu, Michael W.Y. Chan, Alfred S.L. Cheng, Brigette B.Y. Ma, Jun Yu, Kwok Wai Lo, Ka Fai To, Wei Kang

**Affiliations:** 1 Department of Anatomical and Cellular Pathology, Prince of Wales Hospital, The Chinese University of Hong Kong, Hong Kong SAR, China.; 2 Institute of Digestive Disease, State Key Laboratory of Digestive Disease, Li Ka Shing Institute of Health Science, The Chinese University of Hong Kong, Hong Kong SAR, China.; 3 State Key Laboratory of Translational Oncology, Sir Y.K. Pao Cancer Centre, The Chinese University of Hong Kong, Hong Kong SAR, China.; 4 Department of Anaesthesia and Intensive Care and Peter Hung Pain Research Institute, The Chinese University of Hong Kong, Hong Kong SAR, China.; 5 Department of Medicine and Therapeutics, The Chinese University of Hong Kong, Hong Kong SAR, China.; 6 Department of Pathology, Nanfang Hospital and Basic Medical College, Southern Medical University, Guangdong Province Key Laboratory of Molecular Tumor Pathology, Guangzhou, China.; 7 State Key Laboratory of Genetic Engineering, School of Life Sciences, Zhongshan Hospital, Fudan University, Shanghai, China.; 8 Department of Life Science, National Chung Cheng University, Chiayi, Taiwan.; 9 School of Biomedical Sciences, The Chinese University of Hong Kong, Hong Kong SAR, China.; 10 Department of Clinical Oncology, Hong Kong Cancer Institute, The Chinese University of Hong Kong, Hong Kong SAR, China.

**Keywords:** Gastric cancer, YAP, MCM6, PI3K/Akt, therapeutic target.

## Abstract

**Rationale:** Hyperactivation of Hippo-Yes-associated protein (YAP) signaling pathway governs tumorigenesis of gastric cancer (GC). Here we reveal that minichromosome maintenance complex component 6 (MCM6) is a critical transcriptional target of YAP in GC. We aim to investigate the function, mechanism of action, and clinical implication of MCM6 in GC.

**Methods:** The downstream targets of YAP were screened by RNA sequencing (RNA-seq) and microarray, and further validated by chromatin immunoprecipitation PCR and luciferase reporter assays. The clinical implication of MCM6 was assessed in multiple GC cohorts. Biological function of MCM6 was evaluated *in vitro*, in patient-derived organoids, and *in vivo*. RNA-seq was performed to unravel downstream signaling of MCM6. Potential MCM6 inhibitor was identified and the effect of MCM6 inhibition on GC growth was evaluated.

**Results:** Integrative RNA sequencing and microarray analyses revealed MCM6 as a potential YAP downstream target in GC. The YAP-TEAD complex bound to the promoter of MCM6 to induce its transcription. Increased MCM6 expression was commonly observed in human GC tissues and predicted poor patients survival. MCM6 knockdown suppressed proliferation and migration of GC cells and patient-derived organoids, and attenuated xenograft growth and peritoneal metastasis in mice. Mechanistically, MCM6 activated PI3K/Akt/GSK3β signaling to support YAP-potentiated gastric tumorigenicity and metastasis. Furthermore, MCM6 deficiency sensitized GC cells to chemo- or radiotherapy by causing DNA breaks and blocking ATR/Chk1-mediated DNA damage response (DDR), leading to exacerbated cell death and tumor regression. As there are no available MCM6 inhibitors, we performed high-throughput virtual screening and identified purpureaside C as a novel MCM6 inhibitor. Purpureaside C not only suppressed GC growth but also synergized with 5-fluorouracil to induce cell death.

**Conclusions:** Hyperactivated YAP in GC induces MCM6 transcription via binding to its promoter. YAP-MCM6 axis facilitates GC progression by inducing PI3K/Akt signaling. Targeting MCM6 suppresses GC growth and sensitizes GC cells to genotoxic agents by modulating ATR/Chk1-dependent DDR, providing a promising strategy for GC treatment.

## Introduction

Globally, gastric cancer (GC) ranks the fifth most commonly diagnosed cancer and the fourth leading cause of cancer-related mortality, with an estimated 1,089,103 new cases and 768,793 deaths in 2020 1. Surgical resection remains the primary option for GC; however, most GC patients are diagnosed at late stages with lymph node metastasis and not eligible to receive curative surgical treatment 2. Although several therapeutic strategies such as chemotherapy, radiotherapy, and immunotherapy are currently available for advanced GC patients, their survival benefits are still unsatisfactory possibly due to the modest response rate and acquired resistance 3. This situation urges the exploration of molecular mechanisms underlying GC pathogenesis to provide new insights into effective GC treatment.

Emerging evidence has pinpointed the critical role of Hippo signaling pathway in tumor malignancy as it promotes tumorigenesis, metastasis, and therapy resistance 4. Yes-associated protein (YAP), the principal transcriptional coactivator of the Hippo pathway, interacts with transcription factor TEAD to control the expression of downstream targets which are involved in cell proliferation and survival 5. Deregulated YAP is responsible for aggressive tumor properties across diverse human cancers, including GC 6, 7. Previously we revealed that hyperactive or nuclear enriched YAP promoted gastric malignancy and was associated with poor prognosis of GC patients 7. Nevertheless, our understanding of YAP transcriptional targets that contribute to GC growth and therapy resistance is still insufficient. To this end, we performed RNA sequencing (RNA-seq) and microarray in different GC cell lines with or without depletion of YAP which unanimously identified minichromosome maintenance complex component 6 (MCM6) as a potent target of YAP. MCM6 is a highly conserved DNA helicase that form a heterohexameric complex with other members of the MCM family to regulate DNA replication, especially during the initiation and elongation stages 8. MCM6 is found to be overexpressed in several cancer types, including gastrointestinal cancers 9-12, breast cancer 13, neuroblastoma 14, and mantle cell lymphoma 15, and contributes to cancer development 16. High MCM6 expression has been reported to be associated with poor survival of cancer patients 9, 10, 12. However, current knowledge regarding the association between YAP and MCM6 and their functional role in gastric carcinogenesis remains largely unexplored.

Our study identified MCM6 as a novel transcriptional target of YAP in GC. Upregulation of MCM6 in GC was correlated with overexpressed YAP and predicted poor survival of GC patients. YAP-MCM6 axis potentiated GC cell proliferation and metastasis by activating the phosphatidylinositol 3-kinase/protein kinase B (PI3K/Akt) pathway. Depletion of MCM6 abolished GC tumor growth and sensitized GC to standard cytotoxic treatments via suppression of the DNA damage-induced ataxia telangiectasia and rad3-related protein/checkpoint kinase 1 (ATR/Chk1) signaling. Using high-throughput virtual screening, we discovered a novel MCM6 inhibitor, purpureaside C, which exhibited a strong anti-GC effect. Together, our study pinpoints MCM6 as a promising prognostic factor and therapeutic target for GC patients.

## Materials and Methods

### Human tissue samples

Two independent cohorts of GC tissue microarrays were used in this study. Hong Kong cohort containing 264 cases of GC tumor was collected between 1998 and 2006 from Prince of Wales Hospital with complete clinicopathologic and follow-up information (Table S1). Beijing cohort with 162 tumor tissues was collected from Peking University Cancer Hospital, China. The study was approved by the Joint Chinese University of Hong Kong - New Territories East Cluster Clinical Research Ethics Committee (CREC Ref. No.: 2021-083) and Peking University.

### Cell and organoid cultures

Human GC cell lines (AGS, BGC-823, Kato III, MGC-803, MKN1, MKN7, MKN45, NCI-N87, and SGC-7901) were obtained from American Type Culture Collection (ATCC) and RIKEN Cell Bank. Human embryonic kidney cell line HEK293T was purchased from ATCC. All cell lines were cultured in RPMI-1640 (Gibco) or DMEM (Gibco) medium supplemented with 10% fetal bovine serum and penicillin-streptomycin at 37 °C with 5% CO_2_. For organoid culture, GC biopsies were collected from patients undergoing gastrectomy at Prince of Wales Hospital, Hong Kong. Cells were isolated and cultured following previously established protocol 17. Tumor tissues (0.5~1cm^3^) were rinsed, minced, and incubated with collagenase-accutase digestion solution for 1 h at 37 ℃. The digestion was stopped by cold culture medium and the suspension was then filtered through a 70 µm strainer and centrifuged at 400g for 5 min. Next, the cell pellet was mixed with Matrigel (BD Biosciences) to establish a 3D droplet culture model. After the Matrigel solidified, advanced DMEM/F12-based culture medium was added. This study was performed with patients' informed consent and protocol approved by the Ethics Committee as stated above.

### Western blot analysis

The cell and tissue samples were collected and lysed in RIPA buffer supplemented with protease inhibitor cocktail (Roche). Antibodies used are listed in Table S2.

### Chromatin immunoprecipitation (ChIP)

The ChIP assay was performed following the EZ-ChIP™ kit (Merck Millipore) instruction. MGC-803 cells were fixed with 1% formaldehyde at room temperature for 10 min and then glycine was added to quench unreacted formaldehyde for 5 min at room temperature. Cell pellet was resuspended in SDS lysis buffer containing 1X protease inhibitor cocktail (Roche) and sonicated at 4 °C to shear DNA into 200~1000 bps fragments. Immunoprecipitation was performed using anti-YAP antibody (Cell Signaling Technology #14074) and ChIP Protein A/G Magnetic Beads. The crosslink between protein and DNA was reversed at 65 °C. DNA was then purified for real-time quantitative PCR (qPCR) assay. Normalization was performed to the amount of input. Primers sequences were provided in Table S3.

### High-throughput virtual screening (HTVS)

To identify potential MCM6 inhibitors, Schrödinger Maestro version 11.4 was used for structure based HTVS. The X-ray crystal structure of human MCM6 (PDB ID: 6XTX) was obtained from RCSB Protein Data Bank and further processed by the Protein Preparation Wizard module of Schrödinger. MCE Bioactive Compounds Library Plus containing 12,600 compounds was ready for virtual screening after conducting energy optimisation by LigPrep module of Schrödinger. These optimized compounds were then subjected to Virtual Screening Workflow for molecular docking. At first, all compounds were screened in Glide HTVS mode where the top-scored 10% compounds were subjected to the Glide Standard Precision (SP) docking. Subsequently, the top 10% from Glide SP were docked in Glide Extra Precision (XP). The top 15 hit molecules with the highest absolute docking scores were selected as candidates for further experimental validation (Table S4). A higher absolute value of the predicted docking score indicates a stronger binding affinity. The 2D and 3D docking mode were created by PyMol.

### Cellular thermal shift assay (CETSA)

For the cell lysate CETSA 18, cells were harvested and washed once with PBS. The cells were resuspended in PBS supplemented with protease inhibitor cocktail (Roche) and then freeze-thawed three times using liquid nitrogen for complete cell lysis. The cell lysate-containing supernatants were centrifuged at 20,000 g for 20 min at 4 °C to remove cell debris. The lysates were diluted in PBS and divided into two subgroups: one mixed with purpureaside C (150 μM) while the other mixed with empty vehicle. After a 30-min incubation at room temperature, the lysates were aliquoted in 50 μL and each aliquot was heated to the designated temperatures for 3 min (Applied Biosystems) using a thermal cycler, followed by cooling for 3 min at room temperature. The heated lysates were centrifuged at 20,000g for 20 min at 4 °C to collect supernatants with the soluble protein fraction for subsequent western blot analysis.

### Drug combination analysis

GC cells were treated with serial doses of 5-fluorouracil (5-FU) and purpureaside C (under a constant combination ratio). CompuSyn version 1.0 (https://www.combosyn.com/) based on the Chou-Talalay method was used to determine the drug-drug interaction by generating the fraction affected (Fa)-combination index (CI) curves, isobolograms, and Fa-Log (Dose reduction index [DRI]) plots 19, 20. CI and DRI were calculated using the algorithm established by CompuSyn. For each drug combination, CI < 1, CI = 1, and CI > 1 indicate synergism, additive effect, and antagonism, respectively. DRI < 1, DRI = 1, and DRI > 1 indicate favorable dose reduction, no dose reduction, and unfavorable dose reduction, respectively.

### Animal studies

The *Yap/Taz* conditional knockout (*Yap^floxed/floxed^Taz^floxed/floxed^ Ubc-Cre/ERT2*) C57BL/6 mice were established in our previous publication 21. To delete *Yap/Taz,* 6-weeks-old *Yap^floxed/floxed^Taz^floxed/floxed^ Ubc-Cre/ERT2* were injected with tamoxifen (100 mg/kg, i.p., three times a week). Four weeks after tamoxifen administration, successful *Yap^-/-^Taz^-/-^* mice was achieved and confirmed by PCR and western blot. To induce GC, wild-type and *Yap^-/-^Taz^-/-^* mice were served with MNNG-containing drinking water (100 mg/mL) for 14 consecutive days followed by normal drinking water for the next 14 days 22, 23. After administration of MNNG for three cycles, mice were sacrificed on Day 90 for tumor collection. For subcutaneous xenografts, GC (2×10^6^ cells) cells were subcutaneously injected into the right dorsal flanks of 4-weeks-old male Balb/c nude mice. Tumor size was measured every two days by a digital caliper. The mice were sacrificed 16 days after the inoculation, and tumors were harvested and weighted. To investigate the effect of MCM6 on GC metastasis *in vivo*, MGC-803 (1×10^5^ cells) cells transduced with lentiviral carrying shNC, shMCM6-1, and shMCM6-2 were injected into the abdominal cavity of 4-weeks-old male Balb/c nude mice. At the endpoint, peritoneal nodules formed were examined. For the small molecule combinational administration, 12 mice were implanted with 1×10^5^ MGC-803 cells in the abdominal cavity. After four days' inoculation, the mice were randomly divided into four groups (n = 3/group) and then treated with DMSO (control), MK-2206 2HCI (50 mg/kg/day by oral gavage), verteporfin (10 mg/kg by intraperitoneal injection every other day), or MK-2206 2HCI plus verteporfin respectively for consecutive 24 days. For the combined effect of shMCM6 and 5-FU, 1×10^6^ cells MGC-803 cells with or without MCM6 knockdown were injected subcutaneously into the left dorsal flank of four-week-old NSG mice. After 7 days (tumor reaches 50~80mm^3^), we started to treat mice by intraperitoneal injection of 5-FU at 25 mg/kg/every two days for 21 days. The mice were randomly separated into the following four subgroups: (i) PBS with shNC (control); (ii) PBS and shMCM6; (iii) shNC and 5-FU (25 mg/kg); and (iv) shMCM6 and 5-FU. The mice were sacrificed on day 21 after 5-FU treatment. The animal studies were approved by the Animal Experimentation Ethics Committee of CUHK (Ref. No. 21-013-NSF). The animal studies were conducted under the approval of the Animal Experimentation Ethics Committee of CUHK (Ref. No. 21-013-NSF).

### RNA sequencing

Total RNA from GC cells treated as indicated were sequenced on Hiseq-PE150 (Novogene). Reads were quality-checked by FastQC (version 0.11.9) and mapped onto the human reference (GRCh38 with gene annotations GENCODE version 30) by HISAT2 (version 2.1.0) with the default options. The number of reads mapped to each of genes was counted by using featureCount (version 1.6.4). Gene expression levels were calculated as FPKM (Fragments per Kilobase of transcript per Million mapped reads) by rpkm method in edgeR. Differentially expressed genes were determined using DESeq2. Functional enrichment analysis was performed to predict the association of altered genes with disease phenotypes using WebGestalt.

Please see Supplementary Materials for more detailed methods.

## Results

### MCM6 is a downstream target of YAP in GC

**Figure 1:**
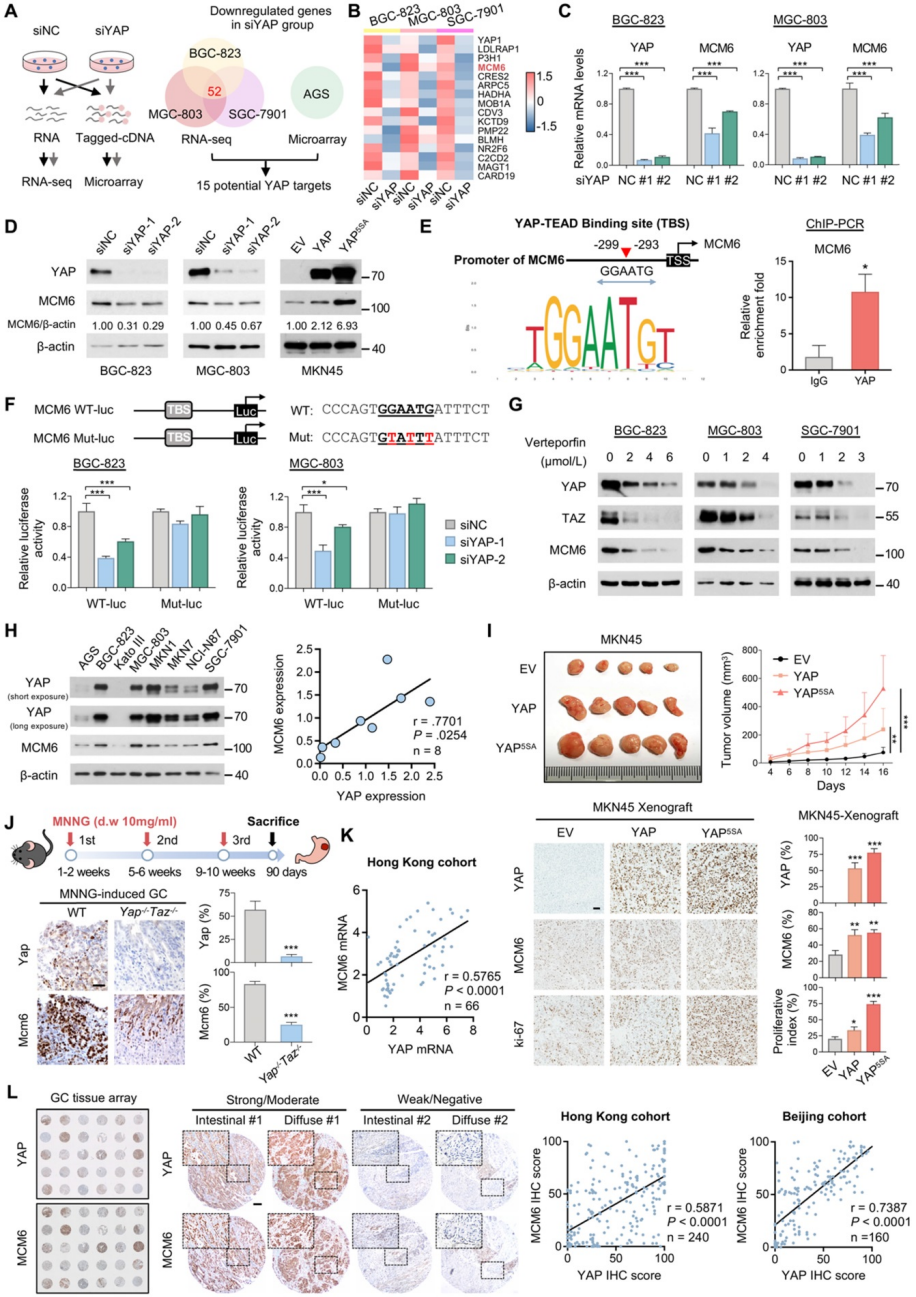
** MCM6 is a transcriptional target of YAP in GC. (A)** Flow chart of RNA-seq and microarray (left). Venn diagram showed overlap of differentially downregulated genes (fold-change ≥ 2) targeted by YAP between RNA-seq and microarray (right). **(B)** Heatmap showed the 15 overlapped genes in GC cells by RNA-seq analysis. **(C-D)** qPCR (C) and western blot (D) analyses of MCM6 in GC cell lines with or without YAP expression modulated. **(E)** Schematic representation of the putative YAP-TEAD binding site on MCM6 promoter region predicted by JASPAR (left). ChIP-PCR confirmed the binding of YAP-TEAD complex to MCM6 in MGC-803 cells (right). TSS, transcription start site. **(F)** Sequences of luciferase reporters carrying wild-type (WT-luc) or mutant (Mut-luc) MCM6 promoter region (top). Luciferase reporter assay validated the interaction between YAP-TEAD complex and MCM6 (bottom).** (G)** Western blot analysis of GC cells treated with verteporfin for 48 h. **(H)** Western blot analysis of YAP and MCM6 in a panel of GC cell lines (left) and measurement of correlation (right).** (I)** Image and growth curves of MKN45 xenograft (top). IHC analysis of YAP, MCM6, and ki-67 in MKN45 xenografts (bottom). Scale bar, 20 μm. **(J)** Schematic diagram of MNNG chemically-induced GC model (top). IHC analysis of Yap and Mcm6 in MNNG-induced GC tumors from wild-type and stomach-specific Yap/Taz-knockout (*Yap^-/-^Taz^-/-^*) mice (bottom). Scale bar, 20 μm. d.w., drinking water. **(K)** Pearson correlation analysis of YAP and MCM6 mRNA expression in Hong Kong GC cohort. **(L)** Representative images of GC cases with strong/moderate or weak/negative expression of YAP and MCM6. Scale bar, 100 μm. Correlation of YAP and MCM6 IHC scores in GC microarrays from Hong Kong and Beijing cohorts. Error bars in C, E, F, I, and J represent mean ± standard deviation. *P < 0.05; **P < 0.01; ***P < 0.001; analysis of variance test (ANOVA) (C, F, and I), Pearson r (H, K, and L) or 2-tailed t test (E and J). EV, empty vector; NC, negative control.

We first verified the oncogenic potential of YAP in GC. As expected, silencing of YAP inhibited proliferation, migration, and invasion of GC cells, whereas ectopic expression of wild-type YAP or constitutively active YAP (YAP^5SA^) exerted the opposite effect (Figure S1A-F). To profile YAP target genes, four GC cell lines with or without YAP knockdown were selected for RNA-seq (BGC-823, MGC-803, and SGC-7901) and microarray (AGS) (Figure 1A). Overlap of differential downregulated genes (fold-change ≥ 2) in these GC cells identified 15 candidates targeted by YAP (Figure 1A-B). Among them, MCM6 was further chosen as it was one of the top-ranked genes that exhibited significantly higher expression in GC compared with paired adjacent normal tissues from TCGA (n = 32, *P* < 0.001). MCM6 belongs to evolutionally and functionally conserved MCM family of DNA helicase. This observation was confirmed by qPCR and western blot showing that knockdown of YAP led to a significant decrease in MCM6 mRNA and protein levels in GC cells (Figure 1C-D). Moreover, overexpression of wild-type YAP or YAP^5SA^ increased MCM6 protein expression (Figure 1D), corroborating that MCM6 is a downstream target of YAP in GC.

### YAP-TEAD transcriptionally induces MCM6 expression in GC

The TEAD family of transcription factors is responsible for YAP-induced gene expression with a consensus binding sequence 'GGAATG' 24. Analysis of MCM6 promoter region (-500bp, +50bp) by JASPAR revealed the presence of potential YAP-TEAD binding sites (Figure 1E). The binding of YAP-TEAD complex to MCM6 promoter region was further confirmed in YAP-overexpressing MGC-803 cells by ChIP-PCR assay (Figure 1E). We next assessed whether the binding of YAP-TEAD complex affects MCM6 expression by constructing luciferase reporters carrying wild-type MCM6 promoter region (WT-luc) or a mutant variant (Mut-luc) (Figure 1F). We found that knockdown of YAP significantly inhibited the luciferase activity of MCM6 reporter in both BGC-823 and MGC-803 cells, whereas no difference was detected in cells transfected with the mutant reporter (Figure 1F). Consistent with these observations, pharmacological disruption of the YAP-TEAD interaction by verteporfin also suppressed YAP and MCM6 protein expression in a dose-dependent manner (Figure 1G). These results demonstrate that YAP induces MCM6 transcription in GC.

We next examined this regulatory relationship by analysing the association between YAP and MCM6 expression. We found YAP was positively correlated with MCM6 expression at both mRNA and protein levels in a panel of GC cell lines (Figure 1H and Figure S2A). In line with this, a strong association between YAP and MCM6 expression was observed in *in vivo* MKN45 xenograft and MNNG-induced GC specimens (Figure 1I-J). Ectopically expressed wild-type YAP or YAP^5SA^ significantly promoted MKN45 subcutaneous xenograft growth (Figure 1I). Immunohistochemistry (IHC) analysis revealed that MCM6 expression was strongly induced in YAP-overexpressing GC tumors together with increased proliferation marker Ki-67 expression (Figure 1I). On the other hand, knockout of Yap/Taz attenuated the inducible effect of MNNG on gastric tumorigenesis using conditional Yap/Taz knockout mice model 21, where tumors developed on MNNG-treated *Yap^-/-^Taz^-/-^* mice expressed significantly less Mcm6 compared to those from WT mice (Figure 1J). Furthermore, the positive association between YAP and MCM6 expression was confirmed in GC samples collected in-house by qPCR assay (Figure 1K), TCGA- STAD dataset (Figure S2B), as well as GC tissue microarrays from Hong Kong and Beijing cohorts by IHC staining (Figure 1L). Nevertheless, knockdown of MCM6 did not alter expression of YAP and YAP target Cyr61 in GC cell lines (Figure S2C), suggesting that there is no feedback loop between YAP and MCM6 in GC. Collectively, our data pinpoint MCM6 as a novel transcriptional target of YAP in GC.

### MCM6 is frequently overexpressed in human GC and indicates a poor prognosis

**Figure 2:**
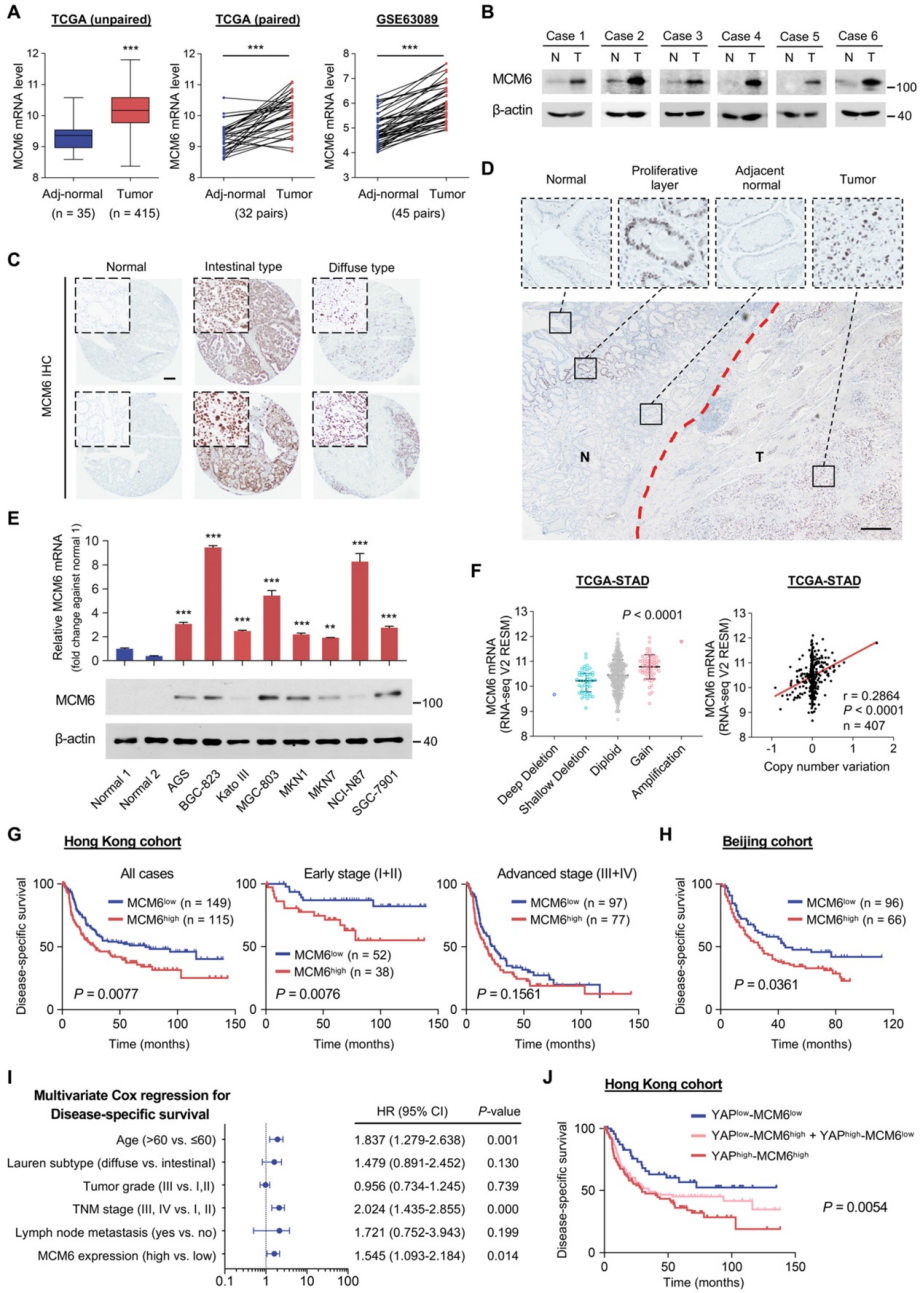
** MCM6 is frequently overexpressed in human GC and indicates a poor prognosis. (A)** Analyses of MCM6 mRNA levels from TCGA and GSE63089 GC datasets. **(B)** Western blot analysis of MCM6 in paired GC tissues. N, adjacent non-tumor; T, tumor.** (C)** Representative IHC images of MCM6 in normal stomach and intestinal-/diffuse-type GC. Scale bar, 100 μm. **(D)** Representative IHC image of MCM6 in tumor (T) and adjacent non-tumor (N) regions. Scale bar, 100 μm. **(E)** qPCR (top) and western blot (bottom) analyses of MCM6 in two normal stomach tissues and a panel of GC cell lines. **(F)** Correlation analysis of MCM6 mRNA level and its DNA copy number in TCGA-STAD dataset.** (G-H)** Kaplan-Meier curves for disease-specific survival of GC patients from Hong Kong (G) and Beijing (H) cohorts. **(I)** Multivariate Cox regression analysis for independent prognostic factors of GC. **(J)** Kaplan-Meier curves for disease-specific survival of each annotated group. Error bars in A, E, and F represent mean ± standard deviation. **P < 0.01; ***P < 0.001; 2 tailed t test (A), paired t test (A), analysis of variance test (ANOVA) (E and F), Pearson r (F) or log-rank test (G, H, and J). CI, confidence interval; TCGA-STAD, The Cancer Genome Atlas-Stomach Adenocarcinoma.

We went further to characterize MCM6 expression in human GC. Using TCGA-STAD dataset, MCM6 mRNA levels were significantly upregulated in GC tumors (n = 415) as compared to adjacent non-tumor tissues (n = 35) (*P* < 0.001; Figure 2A). Similar trends were found in paired GC and non-tumor tissues from TCGA (n = 32, *P* < 0.001; Figure 2A) and a publicly available dataset, GSE63089 (n = 45, *P* < 0.001; Figure 2A). MCM6 protein expression was also increased in GC tumors (Figure 2B). Consistently, higher MCM6 expression was found in both intestinal- and diffuse-type GC tissues compared to normal gastric tissues (Figure 2C), particularly within the tumor area (Figure 2D) by IHC staining. In contrast, MCM6-expressing cells only existed in the isthmus of adjacent non-tumor tissue, a region enrichment of proliferating cells which were PCNA-positive (Figure 2D and Figure S3A-C).

Furthermore, we found MCM6 was highly expressed in eight GC cell lines (AGS, BGC-823, Kato III, MGC-803, MKN1, MKN7, NCI-N87, and SGC-7901) at both mRNA and protein levels, but barely detectable in normal stomach tissues (Figure 2E). MCM6 is therefore frequently upregulated in human GC. In addition, we found that GC samples harboring MCM6 copy number gain exhibited higher MCM6 mRNA expression than those with diploid MCM6 (Figure 2F). A positive correlation between MCM6 copy number variation and its mRNA expression was also determined in GC samples from TCGA (Figure 2F), indicating the upregulation of MCM6 may also be partially due to its copy number gain.

Next, we evaluated the clinical significance of MCM6 in GC. 264 GC patients in our Hong Kong cohort were stratified into MCM6- high and low groups. Although MCM6 protein expression was not associated with clinicopathologic features such as age, gender, Lauren subtype, tumor grade, tumor node metastasis (TNM) stage, and lymph node metastasis (Table S5), Kaplan-Meier survival analysis showed that patients with high-MCM6-expressing GC (43.6%, 115/264) survived for a significantly shortened time than those with low-MCM6-expressing tumors (*P* = 0.0077; Figure 2G). When subdividing patients by TNM tumor stage, high MCM6 protein expression particularly predicted poor prognosis for patients with early (stage I and II) GC (*P* = 0.0076; Figure 2G). The prognostic value of MCM6 was further validated in Beijing cohort wherein high MCM6 protein expression (40.7%, 66/162) was associated with poor disease-specific survival of GC patients (*P* = 0.0361; Figure 2H). Univariate and multivariate Cox regression analyses comprising MCM6 and other risk factors such as age, Lauren subtype, tumor grade, TNM stage, and lymph node metastasis demonstrated that MCM6 was an independent prognostic factor for GC patients (HR, 1.545; 95% confidence interval, 1.093-2.184; *P* = 0.014; Figure 2I and Table S6). Therefore, MCM6 could serve as an independent poor prognostic marker for GC patients. Of note, when we stratified patients into YAP^low^/MCM6^low^ (n = 52), YAP^high^/MCM6^low^ and YAP^low^/MCM6^high^ (n = 97), and YAP^high^/MCM6^high^ (n = 91) subgroups, those with both high YAP and MCM6 expression (YAP^high^/MCM6^high^) had the shortest median disease-specific survival (29.5 months, *P* = 0.0054; Figure 2J). Together, MCM6 is a poor prognostic factor for GC patients.

### MCM6 promotes cell proliferation and metastasis in GC

**Figure 3:**
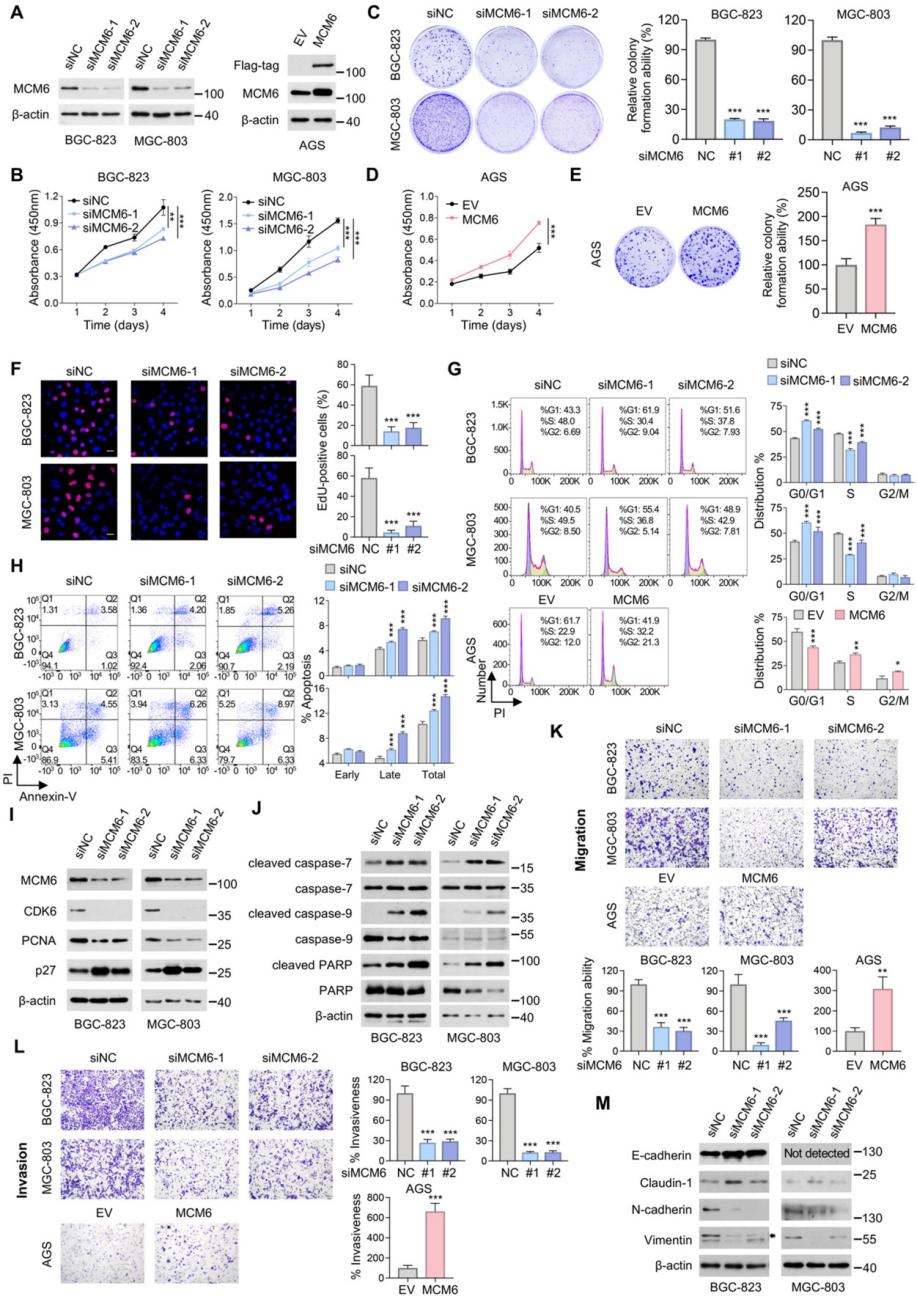
** MCM6 promotes the proliferative and metastatic potential of GC cells. (A)** Western blot validation of MCM6 expression in GC cells with or without MCM6 modulated. **(B-E)** Quantification of cell viability (B, D) and colony formed (C, E) in GC cells with or without MCM6 expression modulated. **(F)** Representative images and quantification of EdU-positive (red) cells in GC cells with or without MCM6 knockdown. DAPI (blue) for nuclei. Scale bar, 20 μm. **(G)** FACS analysis for cell cycle distribution in GC cells with or without MCM6 expression modulated. **(H)** Annexin V apoptosis assay for GC cells with or without MCM6 suppressed. **(I-J)** Western blot analysis of cell cycle- (I) or apoptosis-related (J) proteins in GC cells with or without MCM6 knockdown. **(K-L)** Representative images and quantification of migrated (K) or invaded (L) GC cells with or without MCM6 modulated. **(M)** Western blot analysis of EMT markers in GC cells with or without MCM6 suppressed. Error bars in B-H, K, and L represent mean ± standard deviation. *P < 0.05; **P < 0.01; ***P < 0.001; analysis of variance test (ANOVA) (B-D, F, G, H, K and L) or 2 tailed t test (E, K and L). EMT, epithelial-to-mesenchymal transition; EV, empty vector; NC, negative control; PI, propidium iodide.

To investigate the biological function of MCM6 in GC, we silenced MCM6 by two independent small interfering RNA (siRNA) sequences in high-MCM6-expressing BGC-823 and MGC-803 cells, while overexpressed MCM6 in low-MCM6-expressing AGS cells (Figure 2E). Successful knockdown and overexpression of MCM6 were confirmed at both mRNA and protein levels by qPCR and western blot, respectively (Figure 3A and Figure S4A-B). We observed that silencing of MCM6 suppressed GC cell proliferation and colony formation as compared with the control (Figure 3B-C). Conversely, ectopic expression of MCM6 potentiated the ability of GC cells to proliferate and form colonies (Figure 3D-E). In agreement with these, reduced EdU-incorporated proliferating cells was identified upon MCM6 knockdown (Figure 3F). Moreover, silencing of MCM6 led to G1-phase cell cycle arrest and enhanced cell apoptosis (Figure 3G-H); by contrast, overexpressing MCM6 accelerated the G1-S phase progression (Figure 3G). In parallel, the protein expression of CDK6 and proliferating cell nuclear antigen (PCNA) was downregulated, concomitant with the upregulated p27 after MCM6 knockdown (Figure 3I). On the other hand, increased expression of apoptotic markers such as cleaved-form of caspase-7, caspase-9, and PARP was observed in MCM6-silenced GC cells (Figure 3J) 25.

We next examined whether MCM6 expression affected the metastatic capability of GC. Silencing of MCM6 significantly attenuated the abilities of GC cells to migrate and invade, whereas overexpressing MCM6 yielded the opposite effect (Figure 3K-L). Consistently, knockdown of MCM6 increased expression of epithelial markers (E-cadherin and claudin-1) but decreased expression of mesenchymal markers (N-cadherin and vimentin) as determined by western blot (Figure 3M). These findings collectively support that MCM6 plays an oncogenic role in GC growth and metastasis.

### MCM6 mediates the oncogenic potential of YAP via activating PI3K/Akt signaling

**Figure 4:**
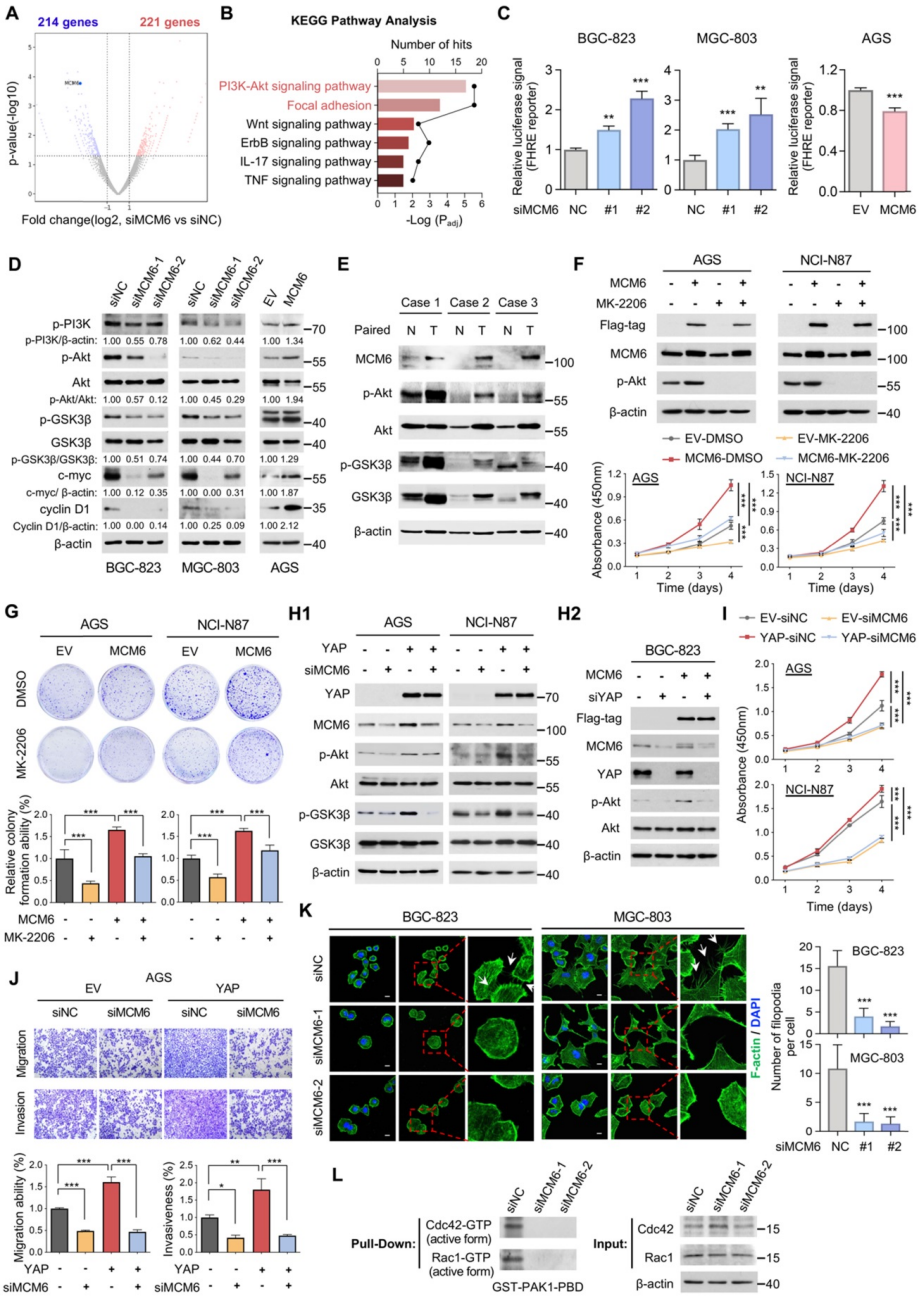
**MCM6 activates PI3K/Akt and focal adhesion signaling in GC. (A)** Volcano plot showed differentially expressed genes from BGC-823 cells treated with siNC vs. siMCM6 by RNA-seq analysis (blue for downregulated; red for upregulated). **(B)** KEGG pathway analysis of altered signaling pathway in BGC-823 cells treated with siNC vs. siMCM6. **(C)** FHRE luciferase reporter assayed the activity of PI3K/Akt pathway in GC cells with or without MCM6 modulated. **(D)** Western blot analysis of GC cells with or without MCM6 modulated. **(E)** Western blot analysis of three pairs of GC clinical samples. N, adjacent non-tumor; T, tumor. **(F-G)** Western blot analysis of GC cells with or without MCM6 overexpressed upon MK-2206 administration (4 μmol/L) (F, top). Quantification of cell viability (F, bottom) and colony formed (G) in GC cells treated as indicated. **(H1-2)** Western blot analysis of GC cells treated as indicated**. (I-J)** Quantification of cell viability (I), migrated or invaded cells (J) in GC cells treated as indicated. **(K)** Immunofluorescence staining of F-actin (green) for the number of filopodia per cells. White arrows indicated the filopodia. DAPI (blue) for nuclei. Scale bar, 10 μm. **(L)** Pull-down assayed the activity of Cdc42 and Rac1 in GC cells with or without MCM6 suppressed. Error bars in C, F, G, I, J and K represent mean ± standard deviation. *P < 0.05; **P < 0.01; ***P < 0.001; analysis of variance test (ANOVA) (C, F, G, I, J and K) or 2 tailed t test (C). EV, empty vector; FHRE, forkhead response element; KEGG, Kyoto Encyclopedia of Genes and Genomes; NC, negative control.

To understand the molecular mechanism underlying the oncogenic function of MCM6 in GC, RNA-seq of BGC-823 cells with or without MCM6 knockdown was conducted. Using a cut-off of ≥ 2-fold-change and a *P*-value < 0.05, a total of 435 differentially expressed genes (221 up and 214 down) were identified upon MCM6 knockdown (Figure 4A). Pathway analysis based on the database for Kyoto Encyclopedia of Genes and Genomes (KEGG) identified several cancer-related pathways that were regulated by MCM6, of which PI3K/Akt and focal adhesion signaling appeared top-ranking (Figure 4B and Figure S5A). From this lead, we sought to investigate the causative relationship between MCM6 and PI3K/Akt signaling activation. By forkhead response element (FHRE) luciferase reporter assay 26, PI3K/Akt pathway was significantly suppressed in MCM6-silenced GC cells, whereas MCM6 overexpression activated PI3K/Akt activity (Figure 4C). Consistently, knockdown of MCM6 reduced the phosphorylation of PI3K (Tyr458), Akt (Ser473), and glycogen synthase kinase-3beta (GSK3β) (Ser9), leading to a decrease in their downstream targets including cyclin D1 and c-myc in GC cells (Figure 4D). On the contrary, ectopic expression of MCM6 showed the opposite effects (Figure 4D). Intriguingly, consistently higher expressions of MCM6, p-Akt, and p-GSK3β were observed in GC samples compared to adjacent normal tissues (Figure 4E). Thus, MCM6 activates PI3K/Akt pathway in GC.

In light of the above findings, we hypothesized that the oncogenic function of MCM6 relies on PI3K/Akt activation. To this end, GC cells with or without MCM6 overexpression were treated with an Akt-specific inhibitor, MK-2206. MK-2206 administration effectively inhibited Akt pathway (Figure 4F and Figure S5B), and abolished MCM6-induced cell proliferation and colony formation in GC cells (Figure 4F-G). Given that Hippo-YAP signaling is well-documented to regulate PI3K/Akt signaling 27, we asked whether MCM6 mediates YAP-induced Akt activation. Expectedly, knockdown of MCM6 attenuated YAP-induced phosphorylation of Akt and GSK3β (Figure 4H1). On the contrary, ectopic expression of MCM6 in YAP-deficient BGC-823 cells reinvigorated PI3K/Akt signaling (Figure 4H2), implying that YAP promotes PI3K/Akt signaling at least partially through MCM6. In addition, deficiency of MCM6 completely abolished YAP-potentiated GC proliferation and metastasis (Figure 4I-J and Figure S5C). Therefore, YAP-MCM6 axis promotes gastric tumorigenicity and metastasis by activating PI3K/Akt signaling.

Focal adhesion pathway was also regulated by MCM6 (Figure 4B and Figure S5A) which is essential for cell migration and invasion 28. By phalloidin staining, which specifically labels cytoskeletal F-actin, we found significantly fewer filopodia in MCM6-silenced cells, in concordance with the weakened metastatic ability by MCM6 knockdown (Figure 4K and Figure 3K-L). Since Cdc42 and Rac1 activation could trigger actin polymerization and filopodia formation 29, their activities were further detected by pull-down assays. Remarkably, MCM6 knockdown suppressed active Cdc42 and Rac1 (Cdc42-GTPase and Rac1-GTPase) expression in BGC-823 cells (Figure 4L). Taken together, MCM6 exerts its oncogenic role via activating PI3K/Akt and focal adhesion signaling in GC.

### MCM6 promotes GC growth and metastasis in patient-derived organoids and xenograft models

**Figure 5:**
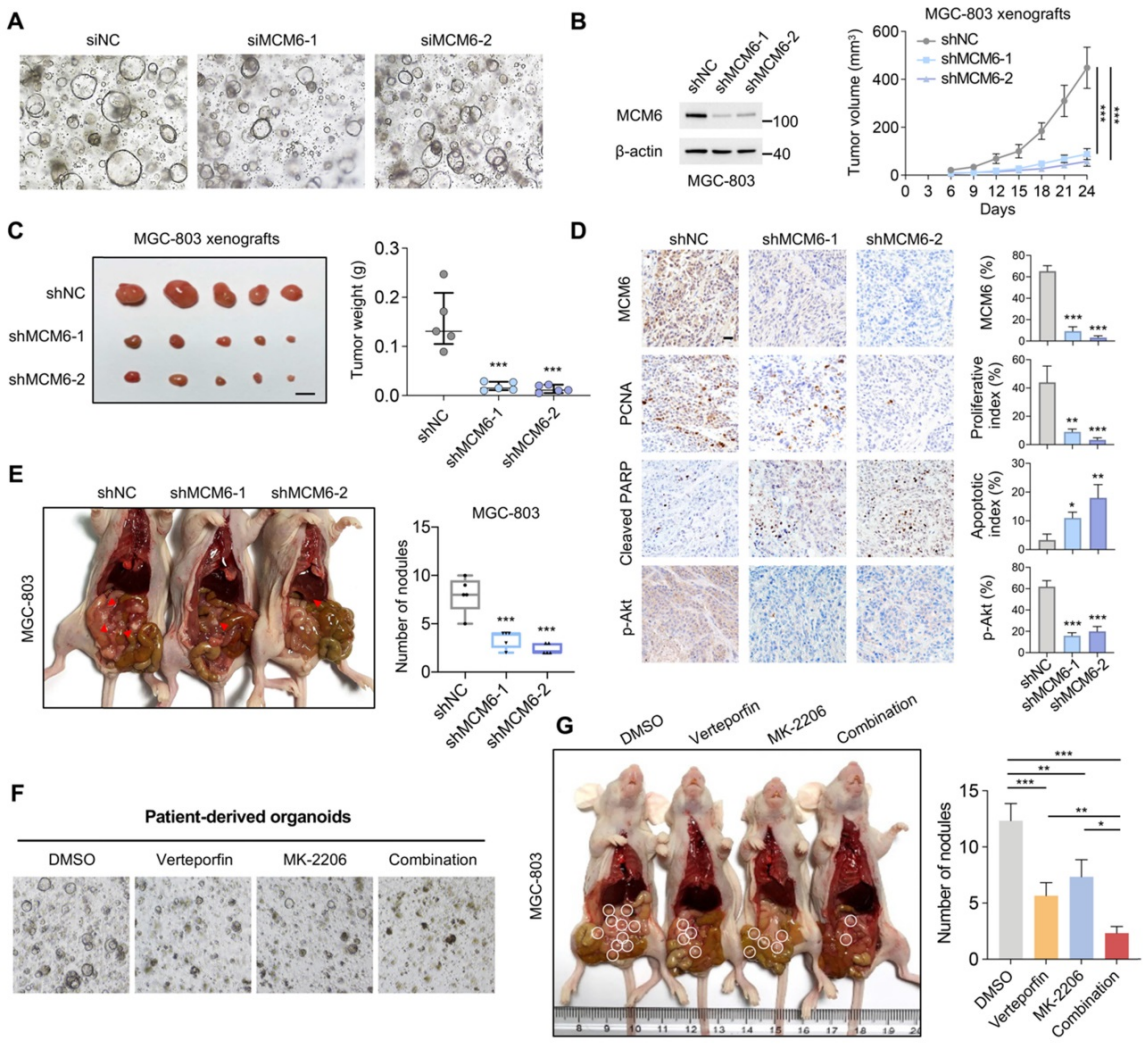
** MCM6 promotes GC growth and metastasis in patient-derived organoids and *in vivo*. (A)** Representative images of patient-derived organoids with or without MCM6 knockdown.** (B)** Growth curve of MGC-803 xenografts in each annotated group (n= 5/group). **(C)** Image of resected MGC-803 xenografts (left) and quantification of tumor weights (right) in each annotated group (n= 5/group). Scale bar, 0.5 cm. **(D)** Representative IHC images and quantification of MCM6, PCNA, cleaved PARP, and p-Akt (Ser473) in MGC-803 xenografts. Scale bar, 20 μm. **(E)** Image and quantification of peritoneal nodules developed in each annotated group (n= 5/group). **(F)** Representative images showed the number and size of GI organoid treated as indicated.** (G)** Image and quantification of peritoneal nodules developed in each annotated group (n= 3/group). Error bars in B and C represent mean ± standard error of the mean and median with interquartile range, respectively. Error bars in D, E, and G represent mean ± standard deviation. *P < 0.05; **P < 0.01; ***P < 0.001; analysis of variance test (ANOVA) (B, C, D, E, and G). NC, negative control.

We further investigated the pro-tumorigenic effect of MCM6 *ex vivo* and *in vivo*. In patient-derived organoids, knockdown of MCM6 inhibited the growth of patient-derived organoid (Figure 5A). MGC-803 cells stably expressing shMCM6 or control cells were subcutaneously injected into the right dorsal flanks of nude mice (Figure 5B). In line with *in vitro* findings, knockdown of MCM6 significantly inhibited tumor growth *in vivo* (*P* < 0.001; Figure 5B). The tumor weight of MGC-803-shMCM6 groups was decreased compared to the control group upon tumor harvesting (*P* < 0.001; Figure 5C). IHC staining of MGC-803 xenografts showed a remarkable decrease of MCM6 and PCNA (cell proliferation marker) expression but increase of cleaved PARP (apoptotic marker) expression in MGC-803-shMCM6 groups (Figure 5D). Furthermore, the expression of p-Akt was reduced in tumors after MCM6 depletion (Figure 5D), which was consistent with our *in vitro* observations (Figure 4D), reinforcing the evidence that MCM6 promotes Akt signaling in GC.

We next studied the effect of MCM6 on GC metastatic ability using the peritoneal metastasis mouse model. GC cells with or without shMCM6 were injected into the peritoneal cavity of nude mice. Results showed that knockdown of MCM6 evidently suppressed the peritoneal dissemination as exemplified by fewer tumor nodules on the peritoneal surfaces in shMCM6 groups as compared with the control group, in agreement with the MCM6-induced epithelial-to-mesenchymal transition (EMT) phenomenon (Figure 5E and Figure 3K-M).

Akt signaling is an attractive target for cancer therapy. We therefore proposed that combination of YAP-MCM6 and Akt inhibition might achieve a better therapeutic effect in GC. To this end, verteporfin, a suppressor of the YAP-TEAD complex, and MK-2206, a highly selective inhibitor of Akt, were administrated to GC organoid and xenograft models. As shown in Figure 5F, co-treatment of verteporfin and MK-2206 markedly reduced both organoid number and size compared to verteporfin or MK-2206-treated alone. In nude mice, the combination of verteporfin and MK-2206 resulted in more significant suppression of peritoneal metastasis than single drug administration (Figure 5G). Collectively, our findings demonstrated that hyperactive YAP enhances MCM6 transcription which in turn triggers the activation of PI3K/Akt signaling cascades to facilitate GC growth and metastasis.

### MCM6 loss sensitizes GC to genotoxic anticancer agents via ATR/Chk1 inactivation

**Figure 6:**
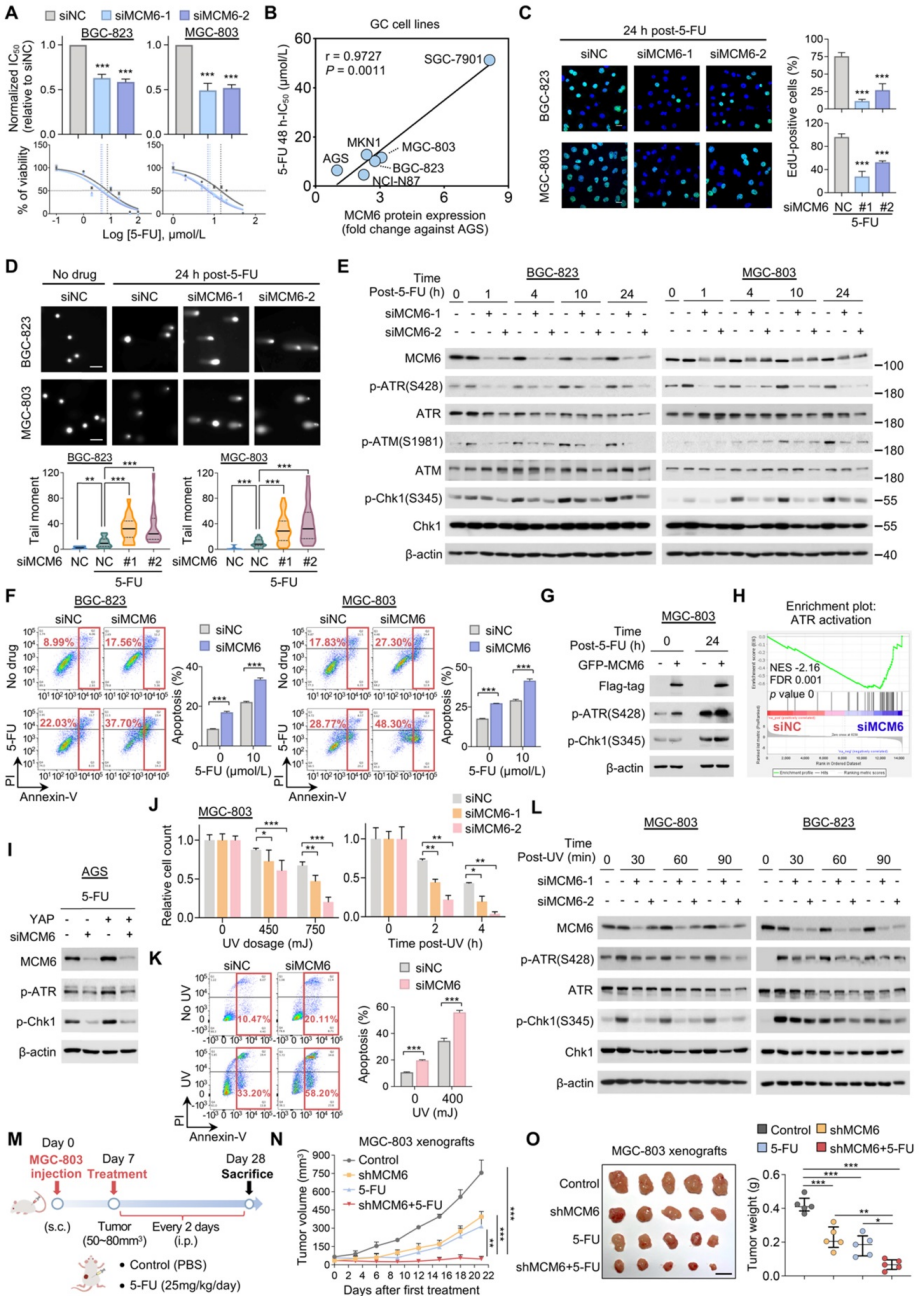
** MCM6 loss sensitizes GC to genotoxic anticancer agents via ATR/Chk1 inactivation. (A)** Determination of 48 h-IC_50_ values of 5-FU in GC cells with or without MCM6 knockdown by cell counting kit-8 (CCK8) assay. **(B)** Pearson correlation analysis of MCM6 protein expression and corresponding 5-FU IC_50_ value in GC cell lines. **(C)** Representative images and quantification of EdU-positive (green) cells after 5-FU administration for 24 h. DAPI (blue) for nuclei. Scale bar, 20 μm. **(D)** Comet assay on GC cells with or without MCM6 knockdown 24 h after 5-FU treatment (10 μmol/L). Scale bar, 100 μm. **(E)** Western blot analysis of GC cells with or without MCM6 knockdown, collected at indicated time points after 5-FU treatment (10 μmol/L). **(F)** Annexin V apoptosis assay for GC cells following a 24 h 5-FU administration. **(G)** Western blot analysis of MCM6-silenced MGC-803 cells and the same cells transfected with ectopic GFP-MCM6 vector upon 5-FU administration (10 μmol/L) for 24 h. **(H)** GSEA analysis of RNA-seq data identified MCM6 was highly associated with ATR activation. **(I)** Western blot analysis of AGS cells treated as indicated.** (J)** Quantification of MGC-803 cells with or without MCM6 knockdown exposed to UV at different dosage (top) or at different time points (bottom). **(K)** Apoptosis assay for MGC-803 cells with or without MCM6 knockdown after exposure to UV (450mJ) for 1 h. **(L)** Western blot analysis of GC cells treated as indicated (UV, 450mJ). **(M)** Schematic diagram of the treatment on MGC-803 xenografts in NSG mice. s.c. subcutaneous injection. i.p., intraperitoneal. **(N)** Growth curve of MGC-803 xenografts in each annotated group (n= 5/group).** (O)** Representative xenograft tumors at endpoint (left) and quantification of tumor weights (right). n = 5/group. Scale bar, 1 cm. Error bars in A, C, D, F, J, K, and N represent mean ± standard deviation. Error bars in O represent median with interquartile range. *P < 0.05; **P < 0.01; ***P < 0.001; analysis of variance test (ANOVA) (A, C, D, F, J, K, N, and O) or Pearson r test (B). FDR, false discovery rate; NC, negative control; NES, normalized enrichment score; PI, propidium iodide.

The fact that YAP confers therapy resistance in various cancer types prompted us to investigate the effect of knocking down MCM6, a critical transcriptional target of YAP in GC, on the sensitivity of GC to chemotherapy 30. Four FDA-approved anticancer drugs (5-FU, cisplatin, oxaliplatin, and doxorubicin) were chosen to treat three high MCM6-expressing GC cell lines (Figure 2E). We found that loss of MCM6 significantly potentiated the growth inhibitory effect of 5-FU in GC cells as indicated by an approximately 2-fold decrease of 48-h half-maximal inhibitory concentration (IC_50_) values (Figure 6A and Figure S6A). Nevertheless, little difference was observed in GC cells treated with other drugs (Figure S6B). Notably, a positive correlation between MCM6 protein expression and their corresponding IC_50_ value of 5-FU was observed in a panel of GC cell lines (Figure 6B and Figure S6C), implying the potential of MCM6 as an indicator of 5-FU therapeutic response. Moreover, silencing of MCM6 markedly caused cell proliferation arrest in GC cells upon 5-FU treatment as evidenced by reduced EdU-positive cells (Figure 6C), in agreement with the role of MCM6 in promoting GC growth (Figure 3F).

We next investigated the possible mechanism by which silencing of MCM6 enhances the therapeutic effect of 5-FU on GC suppression. It is worth noting that 5-FU acts principally by inhibiting thymidylate synthetase to reduce thymidine level for DNA replication and repair, and eventually cause DNA damage and cell death 31. Indeed, 5-FU treatment significantly induced DNA damage in GC cells as manifested by significantly increased tail moments in comet assays (Figure 6D). Remarkably, silencing of MCM6 cooperated with 5-FU to induce extremely high levels of DNA breaks inside GC cells (Figure 6D). In response to DNA damage events, a series of DNA damage response (DDR) machinery is stimulated for DNA repair, allowing cells to survive and proliferate 32. As such, phosphorylation of ATR (Ser428), ataxia-telangiectasia mutated kinase (ATM) (Ser1981), and Chk1 (Ser345) were induced in GC cells upon 5-FU treatment, inferring activation of ATR/Chk1 signaling (Figure 6E). However, silencing of MCM6 prevented the 5-FU induced ATR/Chk1 signaling at multiple time points (Figure 6E). As a consequence, siMCM6 knockdown plus 5-FU significantly enhanced cell apoptosis in both BGC-823 and MGC-803 cells compared to siMCM6 or 5-FU treatment alone (Figure 6F). On the other hand, re-expression of MCM6 restored the protein expression of p-ATR and p-Chk1 in MCM6-deficient GC cells (Figure 6G), suggesting MCM6 is required for GC cells survival in the presence of 5-FU through activating ATR/Chk1 pathway. In line with this, GSEA analysis of our RNA-seq data revealed MCM6 expression induced ATR activation (*P* < 0.001; Figure 6H). Intriguingly, we found YAP was capable to promote ATR/Chk1 signaling via MCM6 in GC cells under 5-FU administration (Figure 6I), corroborating that MCM6 is a critical downstream target of YAP in GC.

Likewise, MCM6 loss also sensitized GC cells to UV irradiation. We found that GC cells with MCM6 knockdown became hypersensitive to UV irradiation in a time- and dose-dependent manner (Figure 6J and Figure S6D), showing higher cell apoptotic rates than those exposed to UV irradiation alone (Figure 6K and Figure S6E). Consistently, silencing of MCM6 remarkably diminished UV-induced phosphorylation of ATR and Chk1 at different time points (Figure 6L). Collectively, our findings demonstrated that depletion of MCM6 sensitized GC cells to genotoxic anticancer agents by triggering DNA damage while compromising DNA repair through suppressing ATR/Chk1 checkpoint pathway, thus resulting in the massive accumulation of damaged DNA and consequent cell death.

We next evaluated the anti-tumor effect of the 5-FU plus shMCM6 in MGC-803 xenografts (Figure 6M). We found that shMCM6 or 5-FU treatment alone significantly inhibited tumor growth compared to the control group, whilst their combination resulted in maximal tumor suppression in GC xenografts (Figure 6N-O). Consistently, the lowest tumor weight was observed in the combination group (Figure 6O). All these data underscore the potential of MCM6 as a therapeutic target for GC. Thus, depletion of MCM6 potentiates the efficacy of chemotherapy in GC.

### MCM6 inhibitor purpureaside C exhibits a strong growth inhibitory effect on GC

**Figure 7:**
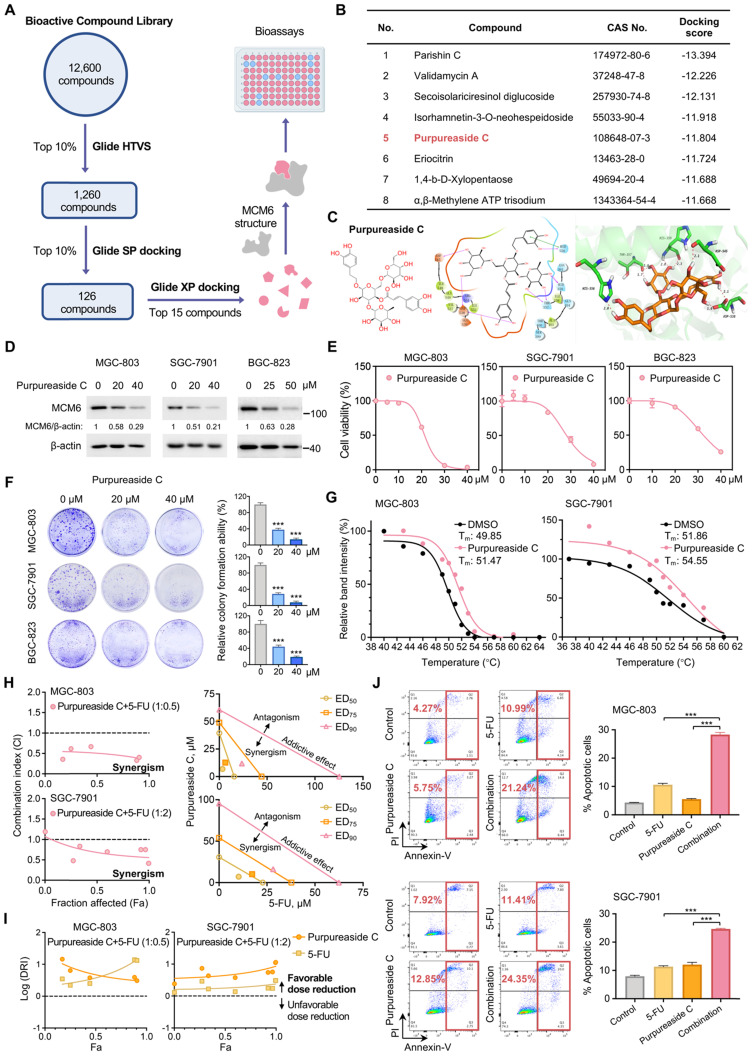
** MCM6 inhibitor purpureaside C exhibits a strong growth inhibitory effect on GC. (A)** Workflow of HTVS for MCM6 inhibitors identification. **(B)** Docking scores of the top 8 candidates obtained from HTVS. **(C)** Chemical structure and *in silico* docking of purpureaside C into the active pocket of human MCM6 protein. **(D)** Western blot analysis of MCM6 expression in GC cells treated by purpureaside C for 48 h. **(E-F)** Quantification of cell viability (E) and colony formed (F) in GC cell lines upon purpureaside C treatment for 48 h. **(G)** CETSA curves for MCM6 protein with or without purpureaside C (150 μmol/L) in GC cell lysates using the Boltzmann sigmoid equation. **(H)** The Fa-CI plots of GC cells exposed to purpureaside C and 5-FU under a constant combination ratio (left). Isobolograms analysis of the combined effect of purpureaside C plus 5-FU at ED_50_, ED_75_, and ED_90_ for GC cells (right). The CI value < 1, = 1, and > 1 indicates synergism, additive effect, and antagonism, respectively. **(I)** The Fa-Log (DRI) plots for the constant ratio combination of purpureaside C and 5-FU. The Log (DRI) value < 0, = 0, and > 0 indicates favorable dose reduction, no dose reduction, and unfavorable dose reduction, respectively. **(J)** Annexin V apoptosis assay for GC cells treated as indicated. Error bars in E, F, and J represent mean ± standard deviation from at least 3 independent experiments. ***P < 0.001; analysis of variance test (ANOVA) (F and J). CI, combination index; DRI, dose reduction index; ED, effective dose; Fa, fraction affected; HTVS, high-throughput virtual screening; PI, propidium iodide; SP, Standard Precision; T_m_, melting temperature; XP, Extra Precision.

Currently, there are no commercially available MCM6 inhibitors. Therefore, we conducted a structure-based high-throughput virtual screening (HTVS) of 12,600 compounds in search of potential MCM6 inhibitors (Figure 7A). Upon sequential SP and XP docking analyses, fifteen compounds with the top-ranked docking scores were selected for further experimental validation (Figure 7A-B and Table S4). Among them, purpureaside C showed the strongest inhibitory effect on MCM6 protein expression. *In silico* docking revealed that purpureaside C binds to the ATP-pocket of MCM6 protein through the seven hydrogen bonds (H-bonds) formed with Asp545, Asp538, Thr357, His359, and His556 (Figure 7C). Western blot indicated that purpureaside C significantly suppressed MCM6 protein expression in GC cells in a dose-dependent manner (Figure 7D). As expected, purpureaside C exhibited remarkable inhibitory effects on BGC-823, MGC-803 and SGC-7901 cells as exemplified by markedly reduced cell viability and colony-forming capacity (Figure 7E-F). We further performed CETSA to determine the interaction between purpureaside C and MCM6 protein. We found that purpureaside C led to substantial shifts of the thermal stability of MCM6 in both MGC-803 and SGC-7901 (Figure 7G), confirming their direct interaction.

We next asked whether inhibition of MCM6 by purpureaside C could also potentiate the anti-tumor effect of 5-FU in GC. Likewise, the combination of purpureaside C and 5-FU exhibited a synergistic effect against GC growth with the CI values less than 1 in two different GC cell lines (Figure 7H). Moreover, their DRI values were more than 1 (Figure 7I), suggesting favorable dose-reduction for each drug in the combination. In line with these, purpureaside C plus 5-FU resulted in the highest percentage of GC cell apoptosis (Figure 7J). Nevertheless, we observed no synergy of purpureaside C and VP in GC (Figure S7A-B).

## Discussion

Exploiting novel cancer therapies is urgent considering a lack of effective treatment options and unsatisfactory clinical benefits for advanced GC patients. Our previous study has pinpointed that aberrant activation of YAP plays a pivotal role in GC progression 7. Here, we further demonstrate for the first time that MCM6 is a critical transcriptional target of Hippo-YAP signaling in GC which mediates the YAP-driven gastric malignancy. MCM6, a DNA helicase that belongs to the highly conserved MCM family, has been implicated in the initiation and elongation of DNA replication 8. We show that YAP binds to the 'GGAATG' motif in the MCM6 promoter region and activates MCM6 transcription. Indeed, a strong positive correlation between YAP and MCM6 expression was observed in a panel of GC cell lines, different GC mice models, and human GC samples, corroborating that MCM6 is a direct downstream target of YAP in GC. Consistent with our findings, the role of YAP/TEAD2 complex in driving MCM6 expression at the transcriptional level was depicted in pancreatic cancer 33. However, no feedback loop between YAP and MCM6 was observed in our study. Intriguingly, a recent publication demonstrated that tumor suppressor CDK5RAP3 may interact with MCM6 to prevent its nuclear translocation of in GC 9. However, our study reveals that MCM6 is predominantly expressed in the nucleus, which is also supported by other publication 34. Thus, the regulatory role of CDK5RAP3 for MCM6 translocation may be context dependent which should be clarified in future.

MCM6 expression is significantly higher in GC tumors compared with non-tumor tissues and predicts poor survival in GC patients in our study, which is same as YAP 7, alluding to the possibility that MCM6 plays an oncogenic role in GC. This is corroborated by both *in vitro* and *in vivo* experiments. We found that MCM6 promoted GC cell cycle progression, concomitant with increased expression of cell cycle regulators such as cyclin D1, CDK6, and PCNA. On the other hand, knockdown of MCM6 induced GC cell apoptosis as exemplified by induction of caspase cascades. Apart from promoting GC proliferation, MCM6 also contributed to the metastatic ability of GC by augmenting the ability of cells to migrate and invade. Knockdown of MCM6 induced epithelial markers expression, e.g., E-cadherin and claudin-1, but inhibited mesenchymal markers expression, e.g., N-cadherin and vimentin. These EMT markers have been well-known to regulate migratory and invasive properties of cancer cells 35. In agreement with our finding, MCM6 has been reported to exert oncogenic functions in liver, breast, and brain cancers 10. Together, MCM6 promotes GC growth and metastasis.

Systematic transcriptome analyses of GC cells revealed that PI3K/Akt signaling pathway was highly associated with MCM6. Accordingly, silencing of MCM6 inhibited PI3K/Akt signaling as evidenced by decreased p-PI3K, p-Akt, and p-GSK3β expression. MCM6 expression was also closely associated with Akt activity in paired GC tissues. Akt acts as a core component of PI3K/Akt signaling cascade which is responsible for coordinating cell response to extrinsic stimuli and contributes to cancer cell proliferation and survival 36-41. Activated Akt prevents cyclin D1 and c-myc from GSK3β-mediated degradation, thereby promoting cell proliferation but inhibiting apoptosis 36-41. All of these are in concordant with the malignant phenotypes driven by MCM6. We showed that blockade of Akt signaling using MK-2206 abrogated the MCM6-driven GC tumorigenicity and metastasis, suggesting that MCM6 exerts its oncogenic functions through activating PI3K/Akt in GC. Notably, YAP is well-documented to regulate PI3K/Akt signaling inside tumors 42, 43. However, YAP failed to induce PI3K/Akt and subsequent cell proliferation and metastasis in MCM6-deficient GC cells, implying that YAP potentiates PI3K/Akt-mediated GC development at least in part through MCM6. In addition to PI3K/Akt pathway, MCM6 also phenocopies the promoting effect of YAP on F-actin polymerization and GC metastasis. Hyperactivated YAP has been reported to upregulate F-actin polymerization and subsequent filopodia formation by controlling Cdc42 activity 28, 29, 44, 45. In agreement, depletion of MCM6 suppresses Cdc42 and Rac1 to impair the formation of invasive protrusion filopodia. Thus, YAP may induce MCM6-Cdc42/Rac1 axis to promote F-actin polymerization, thereby accelerating GC metastasis.

GC patients suffer from poor survival because of the limited therapeutic efficacy. Chemo- and radiotherapy are two available options for advanced GC; however, the survival benefit derived from these treatments is modest due to the emerged resistance. Identification of treatment strategies for improving GC therapy response is therefore urgent and important. Although chemo- or radiotherapy exerts cancer-killing effects by inducing DNA damage, DDR signaling is simultaneously stimulated to coordinate DNA repair process for survival 46. GC resistance to chem- or radiotherapy could be attributed to the activation of DDR pathways 47. The fact that cancer cells with defects in DNA repair are more susceptible to exogenous DNA-targeting agents prompts a strategy of targeting DDR for GC patients who are unresponsive to chemo- or radiotherapy. Our work here revealed that silencing of MCM6 strongly suppressed ATR/Chk1 signaling, a primary direct effector of DNA damage and responsible for cell survival in GC cells upon 5-FU treatment or UV exposure 48. As such, MCM6 depletion together with DNA-damaging agents exacerbated the accumulation of damaged DNA, which further led to massive cancer cell death and tumor regression. On the other hand, re-expression of MCM6 restored ATR/Chk1 signaling in MCM6-deficient GC cells. Therefore, MCM6 is required for the activation of DDR signaling in response to genotoxic anticancer agents. In line with our findings, ATR pathway was activated in several cancers 49, and loss of ATR function could improve cancer response to 5-FU 50. Moreover, we discovered purpureaside C as a potential MCM6 inhibitor, which could directly interact with MCM6 protein to suppress its expression. Strikingly, administration of purpureaside C with 5-FU synergistically suppressed GC growth and induced cell death. Thus, MCM6 is a potential therapeutic target in GC.

To understand how MCM6 activates PI3K/Akt/GSK3β signaling and ATR/Chk1-mediated DNA damage response, we conducted *in silico* protein interaction analysis using the BioGRID data repository (https://thebiogrid.org/). A total of 42 protein are uncovered which exhibit physical interactions with MCM6 including MCM2-7 complex subunits (MCM2-5 and 7) and MCM-binding protein (MCMBP) (Table S7). Among them, SSRP1, a subunit of facilitates chromatin transcription (FACT) complex, is required for the activation of ATR/Chk1 in response to replication stress 51. In addition, USP7 could facilitate Chk1 protein stability by direct deubiquitination, thereby elevating Chk1 levels 52. Furthermore, SSRP1 and USP7 have been identified to promote PI3K/Akt signaling pathway 53, 54. Therefore, MCM6 may bind to SSRP1 and USP7 in the nucleus to activate PI3K/Akt and ATR/Chk1 signaling pathways. Nevertheless, further studies are warranted to prove this hypothesis.

In conclusion, our study uncovers MCM6 as a novel and critical downstream target of YAP in GC. YAP binds to MCM6 promoter to induce its transcription and subsequent PI3K/Akt/GSK3β activation, thus leading to accelerated GC growth and metastasis. Targeting MCM6 by MCM6-siRNA or purpureaside C, our newly identified MCM6 inhibitor, sensitizes GC cells to genotoxic antitumor agents via suppression of DDR. Taken together, MCM6 is a promising prognostic factor and therapeutic target for GC patients.

## Supplementary Material

Supplementary figures and tables.

## Abbreviations

5-FU: fluorouracil
ATCC: American Type Culture Collection
ATM: ataxia-telangiectasia mutated kinase
ATR: ataxia telangiectasia and Rad3-related protein
CETSA: cellular thermal shift assay
ChIP: chromatin immunoprecipitation
Chk1: checkpoint kinase 1
CI: combination index
DDR: DNA damage response
DRI: dose reduction index
EMT: epithelial-to-mesenchymal transition
Fa: fraction affected
FHRE: forkhead response element
GC: gastric cancer
GSEA: gene set enrichment analysis
GSK3β: glycogen synthase kinase-3beta
H-bonds: hydrogen bonds
HR: hazard ratio
HTVS: high-throughput virtual screening
IC_50_: half-maximal inhibitory concentration
IHC: immunohistochemistry
KEGG: Kyoto Encyclopedia of Genes and Genomes
MCM6: minichromosome maintenance complex component 6
MNNG: N-methyl-N'-nitro-N-nitrosoguanidine
RNA-seq: RNA sequencing
PCNA: proliferating cell nuclear antigen
PI3K/Akt: phosphatidylinositol 3-kinase/protein kinase B
siRNA: small interfering RNA
SP: standard precision
STAD: stomach adenocarcinoma
TCGA: The Cancer Genome Atlas
TNM: tumor node metastasis
XP: extra precision
YAP: Yes-associated protein
