# Supplementary material for: MCM6 is a critical transcriptional target of YAP to promote gastric tumorigenesis and serves as a therapeutic target
Source: Theranostics. 2022 Sep 6;12(15):6509–26. doi: 10.7150/thno.75431 (PMC9516235; doi:10.7150/thno.75431)
Supplement: Supplementary file 1 — Supplementary figures and tables. [file thnov12p6509s1.pdf]

# Supplementary Materials for

**MCM6 is a critical transcriptional target of YAP to promote gastric tumorigenesis and serves as a therapeutic target**

Yifei Wang, Huarong Chen, Weixin Liu, Huan Yan, Yihan Zhang, Alvin H.K. Cheung, Jinglin Zhang, Bonan Chen, Li Liang, Zhaocai Zhou, Chi Chun Wong, William K.K. Wu, Michael W.Y. Chan, Alfred S.L. Cheng, Brigette B.Y. Ma, Jun Yu, Kwok Wai Lo, Ka Fai To*, Wei Kang*

*Corresponding authors. E-mail: weikang@cuhk.edu.hk; kfto@cuhk.edu.hk

**This PDF file includes:**

Supplementary Materials and Methods

Figure S1 to S7

Table S1 to S10

References

**SUPPLEMENTARY MATERIAL AND METHODS**

**RNA extraction and real-time quantitative PCR (qPCR).** Total RNA was extracted by RNAiso Plus (Takara). Complementary DNA was synthesized by PrimeScript RT Master Mix (Takara) according to the manufacturer's instructions. qPCR was performed using TB Green Premix Ex Taq (Takara) in QuantStudio 7 Flex Real-Time PCR System (Applied Biosystems). Relative expression differences were calculated using the $2^{-\Delta\Delta Ct}$ method with reference to β-actin. Primers sequences are provided in Table S3.

**Transfection of small interfering RNA (siRNA) and plasmid.** Lipofectamine RNAiMAX (Invitrogen) was used for siRNA transfection, whereas FuGENE HD (Promega) was used for transient plasmid transfection following the manufacturer's instructions. siYAP indicates a mixture of siYAP-1 and siYAP-2. All siRNA used are provided in Table S8.

**Lentiviral production and transduction.** For lentiviral production, lentiviral vector, psPAX2 (Addgene #12260), pMD2.G (Addgene #12259), and lipofectamine 3000 (Invitrogen) were mixed and added to HEK293T cells in 10 cm dish. The lentiviral particle-containing supernatant was harvested at 48 h post-transfection and passed through a 0.45 μm filter. After lentiviral transduction, cells were selected with puromycin (1 μg/mL). Plasmids and short hairpin RNA (shRNA) sequences used are listed in Table S9. pLVX-Flag-YAP-5SA lentiviral vector was kindly provided by Prof. Bin Zhao of Zhejiang University.

**Cell viability and colony formation assays.** Cell viability was measured by cell counting kit-8 (CCK8) from MedChemExpress. Results were presented as the means ± standard deviation (SD) from three independent experiments. For colony formation assay, cells were seeded in 6-well plates at a low density (500 to 1000 cells per well) and cultured in 2ml of complete medium for a week with medium change every two days. At the endpoint, colonies were stained with crystal violet and counted.

**Reagents.** Verteporfin was purchased from Cayman Chemical. Purpureaside C was purchased from MedChemExpress. MK-2206 2HCl, 5-FU, cisplatin, doxorubicin, and oxaliplatin were purchased from Selleck Chemicals.

**Cell migration and invasion assays.** Cell migration and invasion assays were conducted using 24-well Transwell Inserts (Corning) and Matrigel Invasion Chambers (Corning), respectively. Cells suspended in serum-free medium were added to the upper chamber, with 10% FBS-containing medium added to the bottom. After 24 h of incubation, cells that migrated or invaded to the lower surface of membrane were fixed with 4% PFA and stained with crystal violet (Sigma-Aldrich). Images of three randomly selected fields were taken under a 20x objective lens to count cell numbers.

**Cell cycle and apoptosis assays.** For cell cycle assay, cells were harvested and fixed in 70% ethanol at 4 °C overnight, followed by staining with propidium iodide (Sigma-Aldrich). For apoptosis assay, cells were harvested and stained with FITC Annexin V Apoptosis Detection Kit I (BD Biosciences). Samples were analyzed by BD LSRFortessa (BD Biosciences) and FlowJo software.

**EdU proliferation assay.** Cells were seeded onto sterile glass coverslips and overnight was allowed for cells to recover and attach to the coverslip. Afterward, cells were treated with 20 µM of 5-ethynyl-2'-deoxyuridine (EdU) and incubated at 37 °C for 4 h. Cells were stained using EdU Proliferation Kit (iFluor 488) (Abcam) according to the company protocol. Nuclei were stained with DAPI (Invitrogen).

**Luciferase reporter assay.** Forkhead response element (FHRE) luciferase reporter (Addgene, #1789) was used to monitor PI3K/Akt pathway activity [1]. To assess the activity of MCM6 promoter, wild-type or mutant MCM6 promoter region was cloned into the pGL3 luciferase reporter vector. Luciferase reporter was transiently co-transfected with pRL-TK Renilla luciferase vector (Promega #E2241) into GC cells using lipofectamine 3000 (Invitrogen). Luciferase activity was detected by the Dual-

luciferase reporter assay system (Promega). Luciferase reporter activity was presented as a ratio of Firefly: Renilla luminescence.

**Immunohistochemistry (IHC) staining.** Tissue sections were deparaffinized in xylene and rehydrated in graded ethanol, followed by antigen retrieval in Tris-EDTA. The endogenous peroxidase activity was quenched by 3% hydrogen peroxide. The sections were then blocked in fetal bovine serum for 1 h, incubated with primary antibodies at room temperature for 3 h, and incubated with secondary antibodies for 1 h. Signals were detected using DAB (Dako). GC tumors from tissue microarrays were stratified into high and low expression subgroups according to the criteria as follows: for YAP, tumors with ≥ 25% nuclear positive cells were considered as high expression cases, and those with < 25% positive cells were low expression cases; for MCM6, high expression cases were defined as ≥ 50% positive cells, while < 50% were for low expression cases. The IHC score was evaluated by two experienced pathologists. Antibodies used for IHC were listed in Table S10.

**UV irradiation.** Cells were seeded in 6-well plates and cultured for 48 h. After removing the medium, GC cells were exposed to UV irradiation in the UV strantalinker 2400 (Stratagene) and harvested at the indicated time points for western blot or functional assays.

**F-actin labelling.** GC cells were seeded on glass coverslips and fixed in 4% paraformaldehyde permeabilized with 0.1% Triton X-100, and blocked in 3% BSA. Phalloidin FITC Reagent (Abcam) was used to label F-actin. Fluorescent signals were visualized using Carl Zeiss LSM880 confocal microscopy.

**TCGA (The Cancer Genome Atlas) and GSE (Genomic Spatial Event) data analyses.** Gene expression profiling data of MCM6 in GC tumors and adjacent normal samples were analyzed under the stomach adenocarcinoma (STAD) of TCGA (https://xenabrowser.net/) and GSE63089 (https://www.ncbi.nlm.nih.gov/geo/). The

correlation analysis between YAP and MCM6 expression was performed using GEPIA (http://gepia.cancer-pku.cn/).

**Expression microarray.** Total RNA of AGS cells transfected with or without siYAP-1 were extracted and reverse transcribed into cDNA. Synthesized cDNA was then fragmented and labeled using the Quick Amp Gene Expression Labeling kit (Agilent). Next, labeled cDNA targets were hybridized to the Affymetrix GeneChip Human Gene 2.0 ST Array for microarray analysis. The procedure of expression microarray and analysis of the gene expression profiling were conducted by Macrogen, Korea.

**Statistical analysis.** Statistical analyses were performed using GraphPad Prism 8.0.0 (GraphPad, La Jolla, CA), or SPSS version 26.0 (SPSS Inc, Chicago, IL). Results were presented as mean ± SD. The Student's t-test or Mann-Whitney U test was performed to compare the means between two groups, and one-way analysis of variance (ANOVA) with the Tukey post-hoc test was for statistical comparisons between multiple groups. Two-way ANOVA was applied for the combined effect evaluation. The difference in growth rates between groups was determined by repeated-measures ANOVA. Kaplan-Meier survival analysis and log-rank test were performed to evaluate the effect of MCM6 on survival. Clinicopathologic features were analyzed by the Pearson chi-square test or Fisher's exact test. Crude hazard ratios (HR) of disease-specific survival associated with MCM6 expression and other variables were estimated by the univariate Cox proportional hazards regression model and adjusted HR was further estimated by the multivariate Cox regression model. $P < 0.05$ was considered statistically significant.

## SUPPLEMENTARY FIGURES

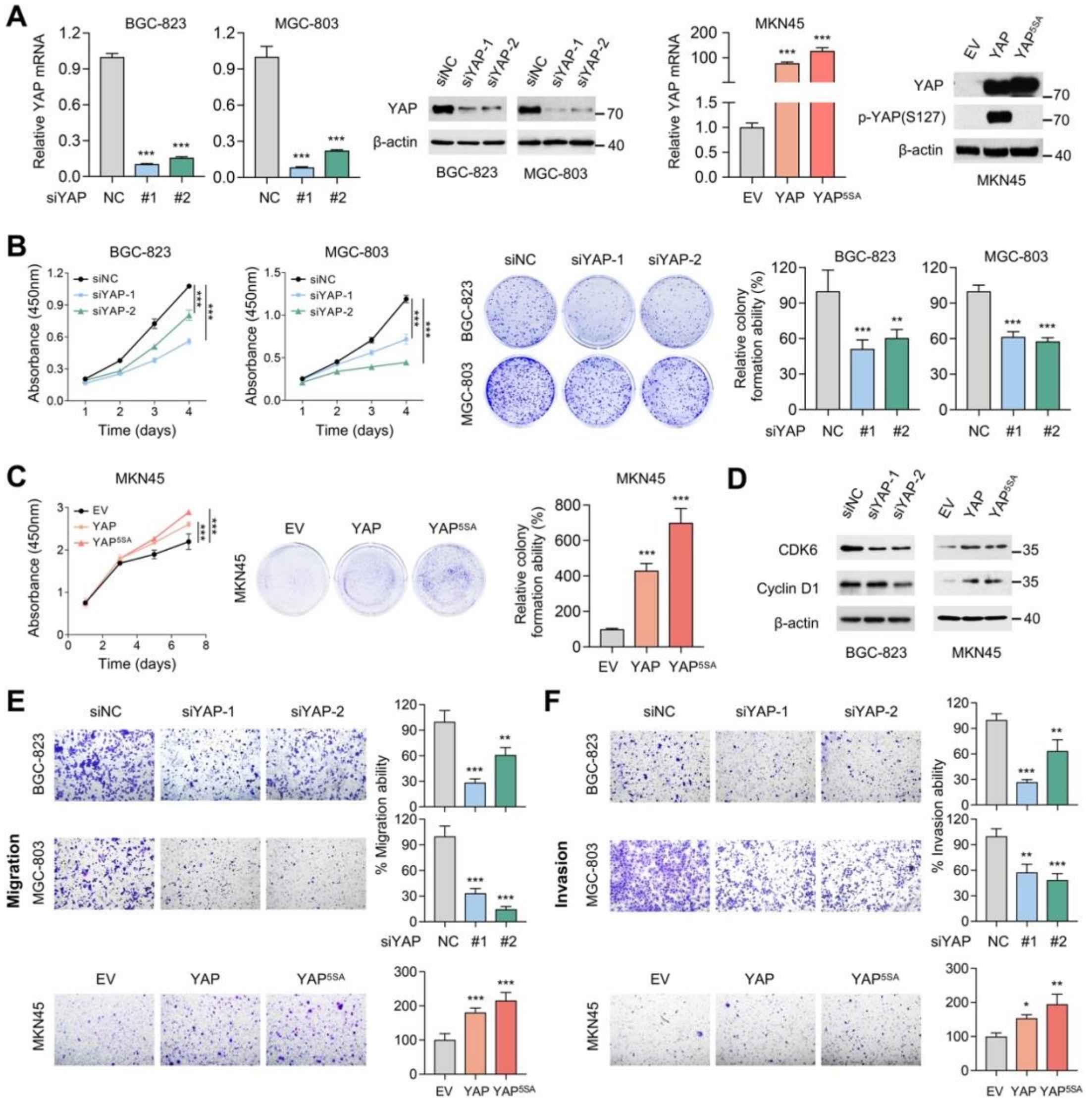


**Figure S1. YAP promotes GC growth and metastasis *in vitro*. (Related to Figure 1)**

**(A)** Two different YAP-siRNAs were used (siYAP-1 and siYAP-2). Quantitative PCR and western blot showed the successful knockdown of YAP expression in BGC-823 and MGC-803 cells. Successful overexpression of wild-type YAP (YAP) and a constitutively active YAP (YAP$^{5SA}$) was also confirmed. **(B-C)** YAP knockdown suppressed the abilities of cells to proliferate and form colony, while overexpressing YAP showed the opposite effects. **(D)** Western blot analysis of CDK6 and cyclin D1 in GC cells with or without YAP knockdown. **(E-F)** Representative images and quantification of GC cells that migrated (E) or invaded (F) with or without YAP modulated. Error bars in A-C, E and F represent mean ± standard deviation. **P* < 0.05; ***P* < 0.01; ****P* < 0.001; analysis of variance test (ANOVA) (A-C, E and F). EV, empty vector; NC, negative control.

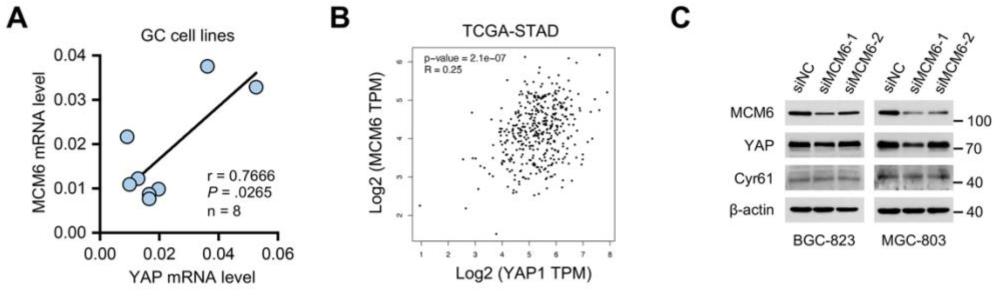


**Figure S2. YAP modulates MCM6 expression in GC. (Related to Figure 1)**

**(A)** Pearson correlation analysis of YAP and MCM6 mRNA expression in eight GC and two normal cell lines. **(B)** Pearson correlation analysis of YAP and MCM6 mRNA levels in GC tumors from TCGA-STAD dataset (http://gepia.cancer-pku.cn/). **(C)** Western blot showed no significant changes of YAP signatures in MCM6-deficient BGC-823 and MGC-803 cells. ***$P$ < 0.001; Pearson r (A and B). NC, negative control; TCGA-STAD, The Cancer Genome Atlas-Stomach Adenocarcinoma.

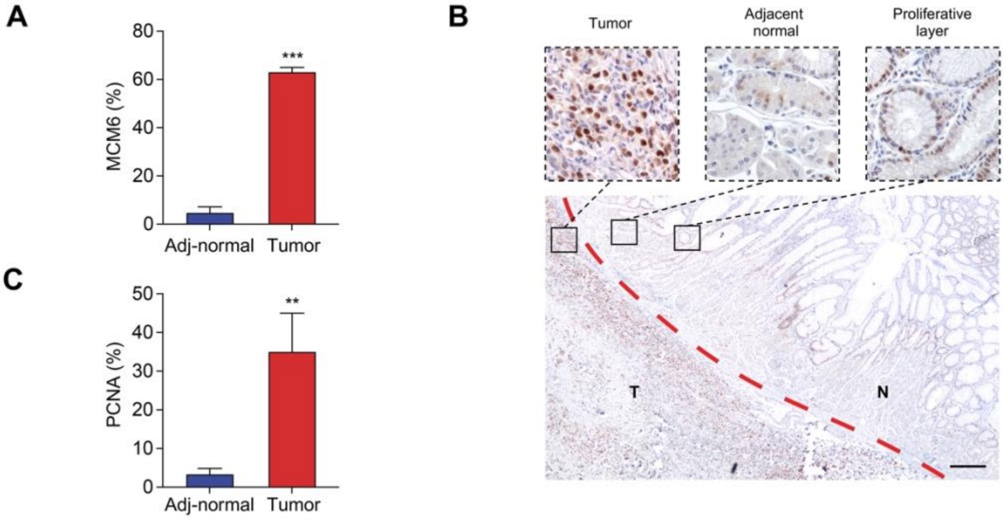


**Figure S3. Expression pattern of PCNA in human GC. (Related to Figure 2)**

**(A)** Quantification of MCM6-positive cells in GC tumors and adjacent normal tissues. **(B-C)** Representative IHC images (B) and quantification (C) of PCNA staining on resected GC tissues showed that PCNA-positive cells had similar expression pattern as MCM6-postive cells did in tumors (T) and adjacent normal (N) tissues. Scale bar, 100 µm. PCNA, proliferating cell nuclear antigen.

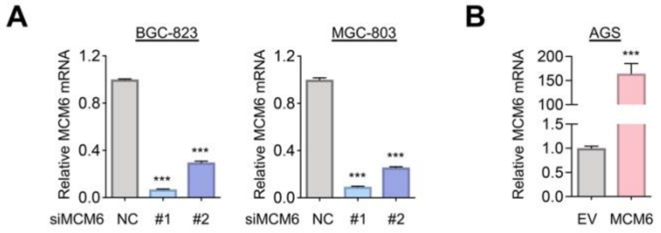


**Figure S4. Knockdown or overexpression of MCM6 in GC cell lines. (Related to Figure 3)**

**(A-B)** qPCR analyses verified successful MCM6 knockdown (A) and overexpression (B) for indicated GC cell lines. Error bars in A and B represent mean ± standard deviation from three independent experiments. ****P* < 0.001; analysis of variance test (ANOVA) (A) or 2-tailed t test (B). EV, empty vector; NC, negative control.

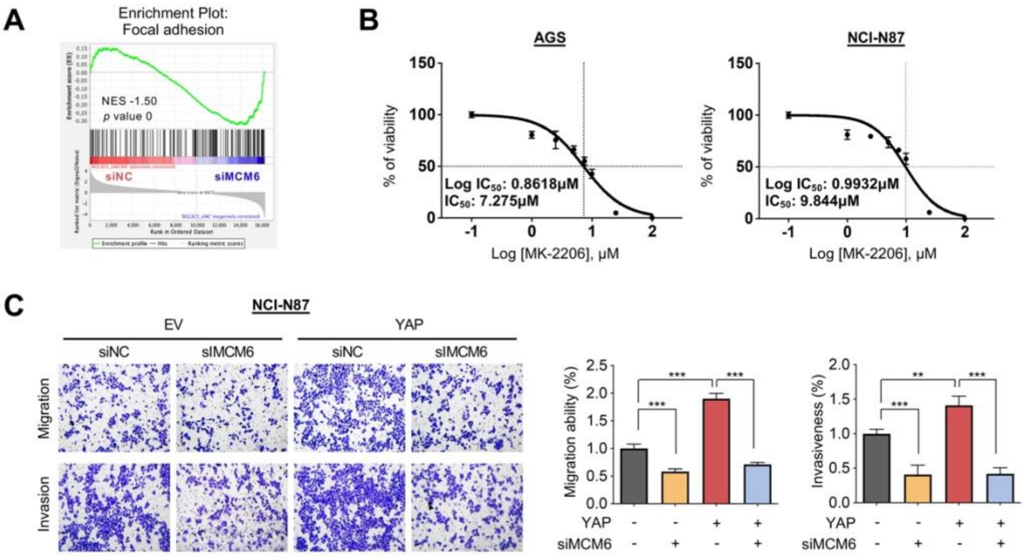


**Figure S5. MCM6 activates PI3K/Akt signaling and focal adhesion in GC cells. (Related to Figure 4)**

**(A)** GSEA analysis identified an enrichment of genes involved in focal adhesion in high-MCM6-expressing BGC-823 cells. **(B)** Determination of 48 h-$IC_{50}$ values of MK-2206 in AGS and NCI-N87 cells by CCK8 assay. **(C)** Representative images and quantification of numbers of NCI-N87 cells that migrated and invaded. MCM6 knockdown partially abolished the tumor metastatic capability of YAP in GC cells. Error bars in B and C represent mean ± standard deviation. $^{**}P < 0.01$; $^{***}P < 0.001$; analysis of variance test (ANOVA) (C). EV, empty vector; GSEA, gene set enrichment analysis; $IC_{50}$, half-maximal inhibitory concentration; NC, negative control; NES, normalized enrichment score.

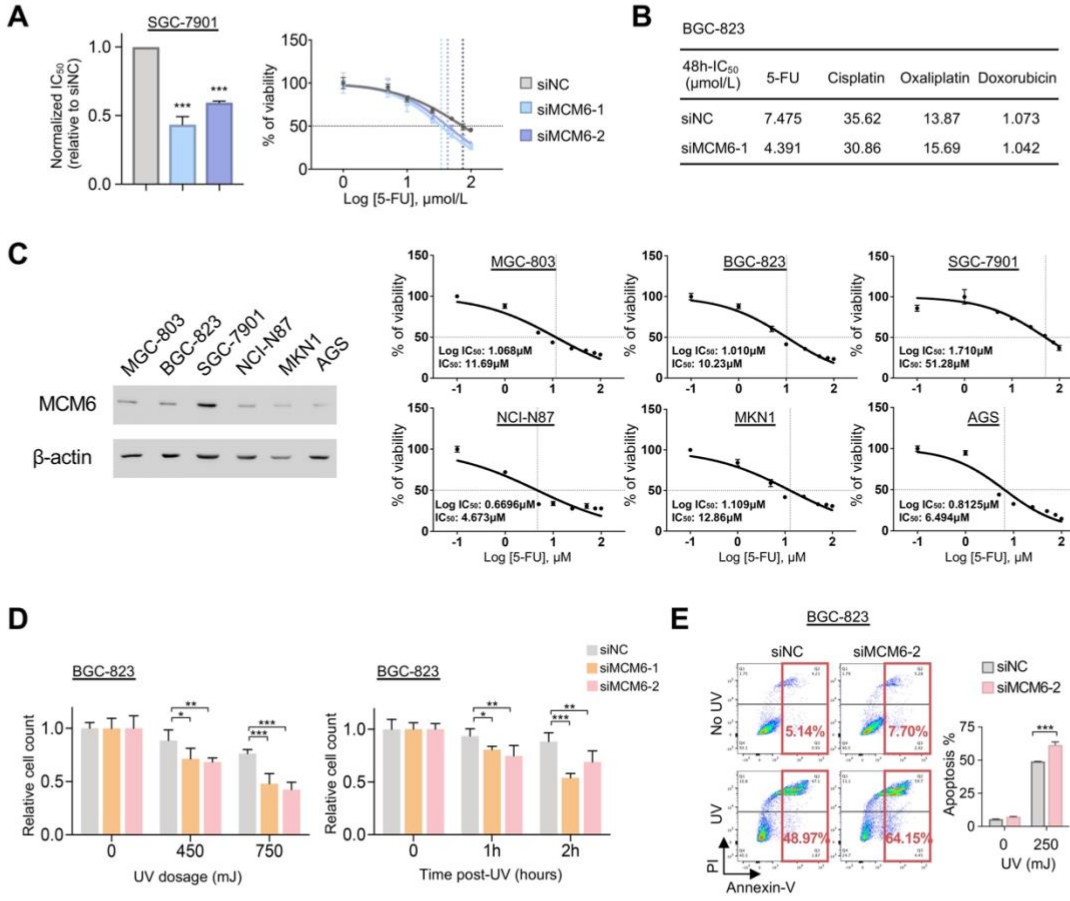


**Figure S6. Knockdown of MCM6 sensitizes cancer therapies in GC. (Related to Figure 6)**

**(A)** Determination of $IC_{50}$ values of 5-FU in SGC-7901 cells with or without MCM6 knockdown. **(B)** $IC_{50}$ values of cisplatin, 5-FU, oxaliplatin, and doxorubicin in BGC-823 cells with or without MCM6 knockdown. **(C)** Western blot analysis of MCM6 protein expression (left) and determination of 48h-$IC_{50}$ values of 5-FU (right) in a panel of GC cell lines. **(D)** Quantification of BGC-823 cells with or without MCM6 knockdown exposed to UV at different dosage or at different time points. **(E)** Apoptosis assay for BGC-823 cells with or without MCM6 knockdown after exposure to UV for 1 h. Error bars in A, D, and E represent mean ± standard deviation. *$P < 0.05$; **$P < 0.01$; ***$P < 0.001$; analysis of variance test (ANOVA) (A, D, and E). 5-FU, fluorouracil; $IC_{50}$, half-maximal inhibitory concentration; NC, negative control; PI, propidium iodide.

**A**

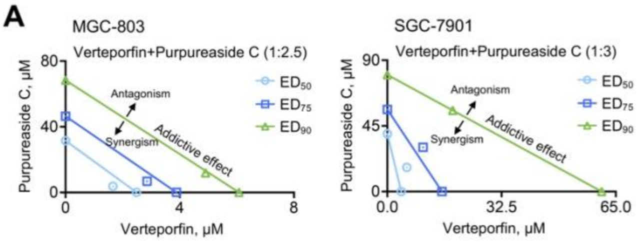


**B**

| Fraction affected (Fa) | Combination index (CI) | |
|---|---|---|
| | MGC-803 | SGC-7901 |
| **0.5** | 0.799 | 1.822 |
| **0.75** | 0.887 | 1.195 |
| **0.9** | 0.985 | 1.001 |

**Figure S7. Combinational treatment of purpureaside C and verteporfin in GC cells. (Related to Figure 7)**

**(A)** Classical isobolograms showed no synergistic effect existed between purpureaside C and verteporfin in both MGC-803 and SGC-7901 cells. **(B)** A table summarized the CI values (calculated using the Chou-Talalay method) of this drug combination when achieved 50% (Fa = 0.5), 75% (Fa = 0.75), and 90% (Fa = 0.90) growth inhibition in MGC-803 and SGC-7901 cells. The CI value <1, = 1, and >1 indicates synergism, additive effect, and antagonism, respectively. CI, combination index; ED, effective dose; Fa, fraction affected.

**SUPPLEMENTARY TABLES**

**Table S1. Characteristics of 264 GC patients in Hong Kong tissue microarray cohort.**

| No. | Sex | Age | Lauren subtype | Tumor grade | TNM stage | LNM | DSS time | DSS status | MCM6 (%) | MCM6 Tumor |
|---|---|---|---|---|---|---|---|---|---|---|
| 1 | male | 52 | diffuse | 2 | IV | positive | 5 | 1 | 0 | low |
| 2 | female | 74 | intestinal | 2 | III | positive | 2.6 | 0 | 0 | low |
| 3 | female | 64 | intestinal | 1 | I | negative | 90 | 0 | 4 | low |
| 4 | male | 82 | diffuse | 3 | III | positive | 10.5 | 1 | 1 | low |
| 5 | male | 69 | intestinal | 2 | II | negative | 30.7 | 0 | 53 | high |
| 6 | male | 74 | intestinal | 2 | I | negative | 70.1 | 0 | 21 | low |
| 7 | female | 52 | intestinal | 2 | IV | positive | 9.4 | 1 | 51 | high |
| 8 | male | 83 | diffuse | 3 | IV | positive | 7.4 | 1 | 82 | high |
| 9 | male | 54 | intestinal | 3 | IV | positive | 83.9 | 0 | 89 | high |
| 10 | male | 72 | intestinal | 2 | III | positive | 48.7 | 1 | 0 | low |
| 11 | female | 55 | diffuse | 3 | II | negative | 87.6 | 0 | 0 | low |
| 12 | female | 67 | intestinal | 3 | II | negative | 89.2 | 0 | 11 | low |
| 13 | male | 58 | intestinal | 2 | III | positive | 55.7 | 0 | 13 | low |
| 14 | male | 35 | diffuse | 3 | III | positive | 67.4 | 0 | 2 | low |
| 15 | male | 79 | intestinal | 3 | II | positive | 15.6 | 0 | 1 | low |
| 16 | female | 75 | diffuse | 3 | IV | positive | 24.7 | 1 | 4 | low |
| 17 | male | 75 | intestinal | 2 | III | positive | 11.2 | 1 | 8 | low |
| 18 | male | 84 | intestinal | 2 | I | negative | 70.8 | 0 | 7 | low |
| 19 | male | 61 | diffuse | 3 | III | positive | 75.8 | 0 | 53 | high |
| 20 | female | 63 | intestinal | 2 | IV | positive | 13.8 | 1 | 23 | low |
| 21 | male | 72 | intestinal | 2 | III | positive | 40 | 1 | 0 | low |
| 22 | male | 53 | diffuse | 3 | IV | positive | 9 | 1 | 0 | low |
| 23 | female | 78 | intestinal | 2 | I | negative | 81.1 | 0 | 3 | low |
| 24 | female | 58 | intestinal | 2 | III | positive | 22.7 | 0 | 51 | high |
| 25 | male | 75 | intestinal | 1 | I | negative | 9 | 1 | 56 | high |
| 26 | male | 49 | diffuse | 3 | IV | positive | 7.5 | 1 | 54 | high |
| 27 | male | 50 | diffuse | 3 | IV | positive | 10.6 | 1 | 0 | low |
| 28 | male | 82 | diffuse | 3 | IV | positive | 17.4 | 1 | 52 | high |
| 29 | female | 73 | diffuse | 3 | IV | positive | 12.3 | 1 | 61 | high |
| 30 | female | 44 | intestinal | 2 | III | positive | 12.6 | 1 | 0 | low |
| 31 | male | 68 | intestinal | 2 | II | positive | 15.3 | 0 | 0 | low |

| | | | | | | | | | | |
|---|---|---|---|---|---|---|---|---|---|---|
| 32 | male | 61 | intestinal | 3 | IV | positive | 11.4 | 1 | 11 | low |
| 33 | female | 88 | intestinal | 2 | III | positive | 0.7 | 0 | 18 | low |
| 34 | female | 82 | intestinal | 3 | III | positive | 4.1 | 0 | 62 | high |
| 35 | male | 80 | intestinal | 3 | III | positive | 11.3 | 1 | 59 | high |
| 36 | male | 77 | intestinal | 2 | II | positive | 73.2 | 0 | 31 | low |
| 37 | male | 88 | intestinal | 3 | III | positive | 9.4 | 1 | 72 | high |
| 38 | male | 64 | intestinal | 2 | II | positive | 76.5 | 0 | 54 | high |
| 39 | male | 73 | intestinal | 2 | III | positive | 7.2 | 0 | 33 | low |
| 40 | male | 68 | diffuse | 3 | IV | positive | 1.5 | 0 | 11 | low |
| 41 | male | 64 | diffuse | 3 | IV | positive | 6.7 | 1 | 51 | high |
| 42 | male | 62 | intestinal | 2 | II | positive | 75.4 | 0 | 22 | low |
| 43 | female | 78 | intestinal | 2 | III | positive | 20.3 | 1 | 0 | low |
| 44 | male | 83 | diffuse | 3 | IV | positive | 24.2 | 1 | 58 | high |
| 45 | male | 69 | intestinal | 2 | IV | positive | 3.9 | 1 | 51 | high |
| 46 | male | 75 | intestinal | 2 | I | negative | 56.4 | 0 | 11 | low |
| 47 | male | 74 | diffuse | 3 | III | positive | 2.3 | 0 | 72 | high |
| 48 | female | 66 | intestinal | 3 | III | positive | 43.6 | 1 | 58 | high |
| 49 | male | 59 | diffuse | 3 | IV | positive | 34.1 | 1 | 25 | low |
| 50 | male | 65 | intestinal | 2 | II | positive | 125.8 | 0 | 11 | low |
| 51 | male | 76 | diffuse | 3 | III | positive | 8.4 | 0 | 53 | high |
| 52 | male | 68 | intestinal | 2 | I | negative | 93.6 | 1 | 34 | low |
| 53 | female | 48 | diffuse | 3 | I | negative | 120.4 | 0 | 0 | low |
| 54 | female | 87 | diffuse | 3 | III | positive | 12 | 1 | 59 | high |
| 55 | female | 43 | intestinal | 3 | IV | positive | 6 | 1 | 32 | low |
| 56 | male | 73 | intestinal | 2 | IV | positive | 2 | 1 | 64 | high |
| 57 | male | 73 | intestinal | 2 | II | negative | 1.3 | 1 | 51 | high |
| 58 | male | 58 | intestinal | 3 | I | negative | 100.4 | 0 | 4 | low |
| 59 | male | 76 | diffuse | 3 | IV | positive | 12 | 1 | 0 | low |
| 60 | male | 61 | intestinal | 3 | IV | positive | 8.9 | 1 | 84 | high |
| 61 | female | 77 | diffuse | 3 | IV | positive | 0.8 | 0 | 38 | low |
| 62 | female | 86 | intestinal | 2 | III | positive | 20.1 | 1 | 61 | high |
| 63 | male | 42 | diffuse | 3 | IV | positive | 13.5 | 1 | 52 | high |
| 64 | male | 67 | intestinal | 1 | I | negative | 116.1 | 0 | 31 | low |
| 65 | female | 86 | intestinal | 3 | III | positive | 0.3 | 0 | 2 | low |
| 66 | male | 70 | diffuse | 3 | IV | positive | 2.3 | 0 | 8 | low |
| 67 | male | 54 | intestinal | 3 | II | positive | 114.6 | 0 | 35 | low |

| | | | | | | | | | | |
|---|---|---|---|---|---|---|---|---|---|---|
| 68 | male | 71 | diffuse | 3 | II | negative | 29.2 | 1 | 13 | low |
| 69 | female | 42 | diffuse | 3 | IV | positive | 16.2 | 1 | 63 | high |
| 70 | male | 36 | diffuse | 3 | III | positive | 14.8 | 1 | 4 | low |
| 71 | female | 54 | intestinal | 2 | III | positive | 29.8 | 1 | 27 | low |
| 72 | female | 69 | diffuse | 3 | IV | positive | 11.9 | 1 | 3 | low |
| 73 | male | 63 | diffuse | 3 | III | positive | 55.1 | 0 | 1 | low |
| 74 | male | 72 | intestinal | 3 | III | positive | 10.8 | 0 | 42 | low |
| 75 | female | 38 | diffuse | 3 | IV | positive | 8.3 | 1 | 15 | low |
| 76 | male | 79 | diffuse | 3 | I | negative | 77.1 | 0 | 0 | low |
| 77 | male | 62 | diffuse | 3 | III | positive | 16.2 | 1 | 61 | high |
| 78 | female | 69 | intestinal | 2 | III | positive | 43 | 0 | 3 | low |
| 79 | female | 73 | intestinal | 2 | IV | positive | 6.3 | 1 | 52 | high |
| 80 | female | 50 | diffuse | 3 | I | negative | 102 | 0 | 16 | low |
| 81 | male | 57 | intestinal | 1 | II | positive | 108.8 | 0 | 2 | low |
| 82 | male | 71 | intestinal | 2 | I | negative | 107.3 | 0 | 0 | low |
| 83 | male | 58 | intestinal | 2 | IV | positive | 35.1 | 1 | 7 | low |
| 84 | male | 77 | intestinal | 2 | III | positive | 15.5 | 0 | 11 | low |
| 85 | female | 75 | intestinal | 3 | I | negative | 108.6 | 0 | 3 | low |
| 86 | male | 72 | intestinal | 2 | IV | positive | 1.8 | 0 | 39 | low |
| 87 | male | 77 | intestinal | 1 | I | negative | 106.3 | 0 | 0 | low |
| 88 | male | 54 | intestinal | 3 | III | positive | 0.5 | 0 | 8 | low |
| 89 | male | 84 | intestinal | 2 | I | negative | 45.8 | 0 | 51 | high |
| 90 | female | 50 | diffuse | 3 | III | positive | 32.6 | 0 | 22 | low |
| 91 | female | 62 | intestinal | 3 | IV | positive | 3.8 | 1 | 28 | low |
| 92 | female | 77 | diffuse | 3 | IV | positive | 28.6 | 1 | 3 | low |
| 93 | male | 77 | diffuse | 3 | III | positive | 116.2 | 1 | 0 | low |
| 94 | male | 72 | intestinal | 3 | III | positive | 34.9 | 1 | 5 | low |
| 95 | female | 46 | diffuse | 3 | IV | positive | 5.9 | 1 | 71 | high |
| 96 | female | 64 | intestinal | 2 | III | positive | 9.5 | 1 | 24 | low |
| 97 | female | 67 | intestinal | 3 | IV | positive | 9.3 | 1 | 3 | low |
| 98 | female | 73 | diffuse | 3 | III | positive | 9 | 1 | 43 | low |
| 99 | female | 74 | diffuse | 3 | III | positive | 7.8 | 1 | 54 | high |
| 100 | female | 78 | diffuse | 3 | I | positive | 16.1 | 1 | 92 | high |
| 101 | male | 56 | diffuse | 3 | III | positive | 143.4 | 0 | 52 | high |
| 102 | female | 64 | diffuse | 3 | IV | positive | 7.9 | 1 | 83 | high |
| 103 | male | 70 | diffuse | 2 | IV | positive | 11.7 | 1 | 12 | low |

| | | | | | | | | | | |
|---|---|---|---|---|---|---|---|---|---|---|
| 104 | female | 76 | intestinal | 2 | II | positive | 6 | 1 | 96 | high |
| 105 | female | 83 | intestinal | 2 | III | positive | 19.1 | 1 | 71 | high |
| 106 | male | 72 | intestinal | 3 | III | positive | 17.2 | 1 | 0 | low |
| 107 | male | 48 | intestinal | 3 | II | positive | 133 | 0 | 67 | high |
| 108 | female | 42 | diffuse | 3 | IV | positive | 3.1 | 1 | 52 | high |
| 109 | female | 70 | intestinal | 2 | I | positive | 134.4 | 0 | 29 | low |
| 110 | male | 73 | diffuse | 3 | III | positive | 15 | 1 | 51 | high |
| 111 | male | 66 | diffuse | 3 | III | positive | 22.7 | 1 | 53 | high |
| 112 | male | 82 | intestinal | 2 | III | positive | 24.5 | 1 | 34 | low |
| 113 | female | 81 | diffuse | 3 | IV | positive | 11.1 | 1 | 2 | low |
| 114 | male | 52 | diffuse | 3 | IV | positive | 2.7 | 1 | 19 | low |
| 115 | male | 48 | intestinal | 2 | III | positive | 22 | 1 | 55 | high |
| 116 | male | 77 | diffuse | 3 | III | positive | 76 | 1 | 12 | low |
| 117 | female | 72 | intestinal | 3 | I | positive | 20.6 | 1 | 24 | low |
| 118 | male | 59 | diffuse | 3 | IV | positive | 98.7 | 0 | 5 | low |
| 119 | female | 86 | intestinal | 2 | III | positive | 20 | 1 | 4 | low |
| 120 | male | 65 | diffuse | 3 | III | positive | 116.4 | 0 | 10 | low |
| 121 | male | 69 | intestinal | 2 | IV | positive | 15.3 | 1 | 3 | low |
| 122 | male | 62 | intestinal | 2 | III | positive | 71.4 | 0 | 36 | low |
| 123 | female | 54 | intestinal | 2 | III | positive | 29.8 | 1 | 11 | low |
| 124 | female | 69 | diffuse | 3 | IV | positive | 11.9 | 1 | 17 | low |
| 125 | male | 62 | diffuse | 3 | III | positive | 55.1 | 1 | 56 | high |
| 126 | male | 72 | intestinal | 3 | III | positive | 15.6 | 0 | 38 | low |
| 127 | female | 38 | diffuse | 3 | IV | positive | 8.3 | 1 | 0 | low |
| 128 | female | 73 | intestinal | 2 | IV | positive | 6.3 | 1 | 58 | high |
| 129 | female | 39 | intestinal | 2 | IV | positive | 3.7 | 1 | 1 | low |
| 130 | male | 57 | intestinal | 1 | II | positive | 108.8 | 0 | 11 | low |
| 131 | male | 58 | intestinal | 2 | IV | positive | 35.1 | 1 | 0 | low |
| 132 | male | 77 | intestinal | 2 | III | positive | 15.5 | 0 | 37 | low |
| 133 | female | 62 | diffuse | 3 | IV | positive | 3.8 | 1 | 53 | high |
| 134 | male | 56 | diffuse | 3 | IV | positive | 10.4 | 1 | 8 | low |
| 135 | male | 64 | intestinal | 2 | III | positive | 32.3 | 1 | 51 | high |
| 136 | male | 50 | diffuse | 3 | II | positive | 17.2 | 1 | 9 | low |
| 137 | female | 73 | diffuse | 2 | IV | positive | 72.3 | 1 | 11 | low |
| 138 | male | 42 | intestinal | 3 | II | positive | 20.4 | 1 | 26 | low |
| 139 | male | 76 | intestinal | 1 | II | positive | 99.6 | 0 | 71 | high |

| | | | | | | | | | | |
|---|---|---|---|---|---|---|---|---|---|---|
| 140 | male | 32 | diffuse | 3 | III | positive | 58.6 | 1 | 35 | low |
| 141 | female | 68 | intestinal | 3 | I | negative | 107.8 | 0 | 26 | low |
| 142 | female | 55 | diffuse | 3 | IV | negative | 106.9 | 0 | 14 | low |
| 143 | female | 53 | diffuse | 3 | IV | positive | 103.1 | 1 | 59 | high |
| 144 | male | 51 | intestinal | 2 | IV | positive | 15.2 | 1 | 88 | high |
| 145 | male | 64 | intestinal | 2 | III | positive | 53.5 | 1 | 33 | low |
| 146 | male | 70 | intestinal | 2 | I | negative | 102 | 0 | 46 | low |
| 147 | male | 58 | intestinal | 2 | III | positive | 96.1 | 0 | 91 | high |
| 148 | male | 82 | intestinal | 2 | I | negative | 39.1 | 0 | 14 | low |
| 149 | female | 50 | diffuse | 3 | IV | positive | 14.4 | 1 | 7 | low |
| 150 | male | 71 | diffuse | 3 | II | negative | 91.2 | 0 | 59 | high |
| 151 | male | 76 | diffuse | 3 | I | negative | 0 | 0 | 27 | low |
| 152 | male | 66 | diffuse | 3 | IV | positive | 4.9 | 1 | 61 | high |
| 153 | male | 37 | intestinal | 2 | II | negative | 90 | 0 | 42 | low |
| 154 | male | 76 | diffuse | 3 | III | positive | 10.6 | 1 | 83 | high |
| 155 | male | 65 | intestinal | 2 | II | positive | 11.8 | 0 | 48 | low |
| 156 | male | 76 | diffuse | 3 | I | positive | 6.9 | 1 | 78 | high |
| 157 | male | 77 | intestinal | 3 | I | positive | 78.1 | 1 | 89 | high |
| 158 | female | 71 | diffuse | 3 | III | positive | 17.9 | 1 | 42 | low |
| 159 | male | 73 | diffuse | 3 | IV | positive | 7.4 | 1 | 75 | high |
| 160 | male | 60 | intestinal | 3 | I | negative | 15.8 | 1 | 86 | high |
| 161 | male | 71 | diffuse | 3 | IV | positive | 40.2 | 1 | 69 | high |
| 162 | male | 35 | diffuse | 3 | I | negative | 88.7 | 0 | 74 | high |
| 163 | male | 69 | diffuse | 3 | IV | positive | 3.9 | 1 | 33 | low |
| 164 | male | 47 | diffuse | 3 | III | positive | 5.1 | 0 | 18 | low |
| 165 | male | 48 | intestinal | 2 | IV | positive | 2.3 | 0 | 55 | high |
| 166 | male | 44 | diffuse | 3 | IV | positive | 12.8 | 1 | 45 | low |
| 167 | male | 61 | diffuse | 3 | IV | positive | 8.8 | 1 | 4 | low |
| 168 | female | 44 | diffuse | 3 | IV | positive | 7.1 | 1 | 96 | high |
| 169 | female | 48 | intestinal | 2 | I | negative | 80.8 | 0 | 54 | high |
| 170 | male | 55 | intestinal | 2 | IV | positive | 52.4 | 1 | 77 | high |
| 171 | male | 82 | intestinal | 2 | IV | positive | 7.8 | 1 | 91 | high |
| 172 | male | 74 | diffuse | 3 | III | positive | 29.5 | 1 | 82 | high |
| 173 | female | 67 | intestinal | 2 | I | negative | 80.6 | 0 | 74 | high |
| 174 | male | 67 | diffuse | 3 | IV | negative | 2.4 | 1 | 7 | low |
| 175 | male | 56 | intestinal | 2 | I | positive | 29.7 | 0 | 82 | high |

| | | | | | | | | | | |
|---|---|---|---|---|---|---|---|---|---|---|
| 176 | male | 61 | diffuse | 3 | III | positive | 7.4 | 1 | 11 | low |
| 177 | female | 57 | intestinal | 3 | I | negative | 15.9 | 0 | 37 | low |
| 178 | male | 66 | intestinal | 3 | II | positive | 1 | 0 | 74 | high |
| 179 | female | 60 | diffuse | 3 | IV | positive | 5.9 | 1 | 100 | high |
| 180 | male | 56 | diffuse | 3 | IV | positive | 78.9 | 0 | 3 | low |
| 181 | male | 65 | intestinal | 3 | I | negative | 79.1 | 0 | 25 | low |
| 182 | female | 73 | diffuse | 3 | III | positive | 19.3 | 1 | 77 | high |
| 183 | male | 69 | diffuse | 3 | IV | positive | 10.3 | 1 | 56 | high |
| 184 | male | 78 | diffuse | 3 | IV | positive | 6.2 | 1 | 67 | high |
| 185 | female | 65 | intestinal | 3 | III | positive | 41.7 | 1 | 100 | high |
| 186 | male | 70 | diffuse | 3 | I | negative | 28.5 | 1 | 55 | high |
| 187 | male | 71 | intestinal | 2 | III | positive | 3.3 | 1 | 51 | high |
| 188 | male | 57 | diffuse | 3 | IV | positive | 55.4 | 1 | 69 | high |
| 189 | male | 65 | diffuse | 3 | IV | positive | 24.9 | 1 | 76 | high |
| 190 | male | 75 | intestinal | 3 | II | positive | 30.7 | 1 | 19 | low |
| 191 | male | 52 | intestinal | 2 | III | positive | 27.5 | 1 | 92 | high |
| 192 | male | 54 | diffuse | 3 | III | positive | 73.3 | 0 | 45 | low |
| 193 | male | 60 | intestinal | 3 | II | positive | 69.4 | 0 | 74 | high |
| 194 | male | 37 | intestinal | 2 | I | negative | 71.2 | 0 | 100 | high |
| 195 | female | 80 | diffuse | 3 | II | negative | 26.6 | 0 | 66 | high |
| 196 | female | 66 | intestinal | 2 | I | positive | 75.2 | 0 | 67 | high |
| 197 | male | 66 | intestinal | 3 | IV | positive | 18.7 | 1 | 81 | high |
| 198 | male | 77 | intestinal | 2 | I | negative | 70.9 | 0 | 57 | high |
| 199 | female | 68 | intestinal | 3 | I | negative | 70.8 | 0 | 52 | high |
| 200 | female | 59 | diffuse | 3 | II | negative | 32.3 | 1 | 3 | low |
| 201 | male | 50 | diffuse | 3 | IV | positive | 9.8 | 1 | 49 | low |
| 202 | female | 75 | intestinal | 2 | I | negative | 51.6 | 1 | 67 | high |
| 203 | male | 52 | diffuse | 3 | IV | positive | 7 | 1 | 88 | high |
| 204 | male | 51 | intestinal | 2 | IV | positive | 20.9 | 1 | 39 | low |
| 205 | male | 56 | intestinal | 2 | IV | positive | 25.9 | 1 | 40 | low |
| 206 | male | 67 | intestinal | 2 | I | negative | 70.4 | 0 | 26 | low |
| 207 | male | 54 | diffuse | 3 | I | positive | 66.2 | 0 | 32 | low |
| 208 | male | 76 | diffuse | 3 | I | positive | 1.8 | 0 | 93 | high |
| 209 | male | 49 | diffuse | 3 | IV | positive | 5.7 | 0 | 4 | low |
| 210 | male | 51 | intestinal | 2 | II | positive | 68.7 | 0 | 92 | high |
| 211 | male | 58 | intestinal | 2 | II | positive | 68 | 0 | 45 | low |

| | | | | | | | | | | |
|---|---|---|---|---|---|---|---|---|---|---|
| 212 | female | 49 | intestinal | 2 | III | positive | 54 | 0 | 93 | high |
| 213 | male | 55 | diffuse | 3 | III | positive | 62.7 | 0 | 72 | high |
| 214 | male | 88 | intestinal | 2 | III | positive | 17.4 | 0 | 46 | low |
| 215 | female | 48 | diffuse | 3 | I | negative | 25.2 | 0 | 43 | low |
| 216 | male | 62 | diffuse | 3 | I | negative | 64.9 | 1 | 61 | high |
| 217 | male | 65 | intestinal | 1 | III | positive | 64.4 | 0 | 77 | high |
| 218 | female | 51 | diffuse | 3 | IV | positive | 20.8 | 1 | 47 | low |
| 219 | female | 49 | diffuse | 3 | III | positive | 62.4 | 0 | 7 | low |
| 220 | female | 61 | intestinal | 2 | II | positive | 42 | 1 | 97 | high |
| 221 | male | 74 | intestinal | 2 | I | negative | 7.4 | 1 | 65 | high |
| 222 | male | 53 | intestinal | 2 | IV | positive | 29.5 | 1 | 92 | high |
| 223 | male | 57 | diffuse | 3 | III | positive | 20.6 | 1 | 13 | low |
| 224 | female | 50 | intestinal | 2 | II | positive | 60.9 | 0 | 11 | low |
| 225 | male | 76 | intestinal | 2 | IV | positive | 2.1 | 1 | 89 | high |
| 226 | male | 38 | diffuse | 3 | III | positive | 27.2 | 1 | 27 | low |
| 227 | male | 82 | diffuse | 3 | III | positive | 9.2 | 0 | 100 | high |
| 228 | male | 56 | intestinal | 2 | IV | positive | 61.1 | 0 | 72 | high |
| 229 | male | 51 | diffuse | 3 | IV | positive | 20.7 | 0 | 31 | low |
| 230 | female | 76 | intestinal | 2 | I | negative | 59.3 | 0 | 60 | high |
| 231 | male | 73 | diffuse | 3 | III | positive | 5.1 | 0 | 31 | low |
| 232 | female | 68 | intestinal | 2 | I | negative | 60.4 | 0 | 15 | low |
| 233 | male | 64 | intestinal | 2 | I | positive | 60.7 | 0 | 71 | high |
| 234 | female | 48 | diffuse | 3 | I | negative | 59.9 | 0 | 51 | high |
| 235 | female | 71 | diffuse | 3 | I | negative | 59.1 | 0 | 42 | low |
| 236 | female | 69 | diffuse | 3 | IV | positive | 6.3 | 1 | 96 | high |
| 237 | male | 52 | diffuse | 3 | IV | positive | 21.2 | 0 | 63 | high |
| 238 | male | 57 | intestinal | 3 | III | positive | 41 | 0 | 92 | high |
| 239 | male | 44 | diffuse | 3 | IV | positive | 47.1 | 0 | 0 | low |
| 240 | male | 77 | diffuse | 3 | III | positive | 0.8 | 1 | 6 | low |
| 241 | male | 52 | diffuse | 3 | IV | positive | 21.7 | 0 | 52 | high |
| 242 | female | 32 | intestinal | 2 | IV | positive | 18 | 0 | 89 | high |
| 243 | female | 48 | diffuse | 3 | IV | positive | 54.5 | 0 | 3 | low |
| 244 | female | 74 | intestinal | 2 | I | negative | 55 | 0 | 93 | high |
| 245 | female | 72 | intestinal | 2 | III | positive | 44.2 | 0 | 24 | low |
| 246 | male | 72 | diffuse | 3 | III | positive | 22.5 | 0 | 11 | low |
| 247 | female | 41 | diffuse | 3 | III | positive | 0.6 | 0 | 65 | high |

| | | | | | | | | | | |
|---|---|---|---|---|---|---|---|---|---|---|
| 248 | male | 65 | intestinal | 2 | I | negative | 50.9 | 0 | 45 | low |
| 249 | male | 60 | diffuse | 3 | I | negative | 55.9 | 0 | 61 | high |
| 250 | female | 72 | diffuse | 3 | IV | positive | 21.8 | 1 | 0 | low |
| 251 | male | 72 | diffuse | 3 | IV | positive | 6 | 1 | 100 | high |
| 252 | male | 70 | intestinal | 2 | III | positive | 51.5 | 0 | 19 | low |
| 253 | male | 48 | diffuse | 3 | III | positive | 51.6 | 0 | 33 | low |
| 254 | male | 62 | intestinal | 2 | I | negative | 139.4 | 0 | 5 | low |
| 255 | male | 63 | diffuse | 3 | III | positive | 15.8 | 0 | 39 | low |
| 256 | male | 51 | diffuse | 3 | I | negative | 138.1 | 0 | 67 | high |
| 257 | male | 61 | diffuse | 3 | II | positive | 135.1 | 0 | 3 | low |
| 258 | male | 54 | intestinal | 3 | III | positive | 70.5 | 0 | 62 | high |
| 259 | male | 72 | intestinal | 2 | I | negative | 91.6 | 0 | 0 | low |
| 260 | female | 77 | diffuse | 3 | IV | positive | 49 | 0 | 0 | low |
| 261 | female | 34 | intestinal | 3 | I | negative | 138.1 | 0 | 42 | low |
| 262 | male | 69 | intestinal | 3 | III | positive | 127.8 | 0 | 98 | high |
| 263 | female | 66 | intestinal | 1 | I | negative | 68.7 | 1 | 58 | high |
| 264 | female | 32 | diffuse | 3 | I | negative | 89.7 | 0 | 11 | low |

DSS, disease-specific survival; LNM, lymph node metastasis.

**Table S2. Antibodies used for western blot.**

| Antibodies | Source | Cat. No. | Working concentration |
|---|---|---|---|
| Rabbit monoclonal anti-MCM6 | Abcam | ab201683 | 1:1000 |
| Mouse monoclonal anti-β-actin | Immunoway | YM3028 | 1:1000 |
| Rabbit monoclonal anti-YAP | Abcam | ab52771 | 1:1000 |
| Rabbit monoclonal (D24E4) anti-YAP/TAZ | Cell Signaling | #8418 | 1:1000 |
| Mouse monoclonal (AE005) anti-FLAG | ABclonal | AE005 | 1:2000 |
| Rabbit monoclonal (92G2) anti-Cyclin D1 | Cell Signaling | #2978 | 1:1000 |
| Mouse monoclonal (DCS83) anti-CDK6 | Cell Signaling | #3136 | 1:1000 |
| Mouse monoclonal (12D10) anti-PCNA | Immunoway | YM3031 | 1:2000 |
| Mouse monoclonal anti-p27 | BD Biosciences | 610241 | 1:1000 |
| Rabbit anti-Cleaved caspase-7 | Cell Signaling | #9491 | 1:1000 |
| Mouse monoclonal (C9) anti-Caspase-9 | Cell Signaling | #9508 | 1:1000 |
| Rabbit anti-Cleaved PARP | Cell Signaling | #9541 | 1:500 |
| Rabbit anti-Caspase-7 | Cell Signaling | #9492 | 1:1000 |
| Rabbit monoclonal (46D11) anti-PARP | Cell Signaling | #9532 | 1:1000 |
| Rabbit anti-phosphorylated YAP (Ser127) | Cell Signaling | #4911 | 1:1000 |
| Rabbit monoclonal (24E10) anti-E-cadherin | Cell Signaling | #3195 | 1:1000 |
| Rabbit anti-N-cadherin | Immunoway | YT2988 | 1:500 |
| Rabbit monoclonal (D21H3) anti-Vimentin | Cell Signaling | #5741 | 1:1000 |
| Rabbit anti-Claudin-1 | Cell Signaling | #4933 | 1:1000 |
| Rabbit anti-phosphorylated PI3 Kinase p85 (Tyr458)/p55 (Tyr199) | Cell Signaling | #4228 | 1:1000 |
| Rabbit anti-phosphorylated AKT (Ser473) | Cell Signaling | #9271 | 1:1000 |
| Rabbit anti-phosphorylated GSK-3β (Ser9) | Cell Signaling | #9336 | 1:1000 |
| Rabbit anti-AKT | Cell Signaling | #9272 | 1:1000 |
| Rabbit monoclonal (27C10) anti-GSK-3β | Cell Signaling | #9315 | 1:1000 |
| Rabbit monoclonal (D84C12) anti-c-Myc | Cell Signaling | #5605 | 1:1000 |
| Rabbit anti-phosphorylated ATR (Ser428) | Cell Signaling | #2853 | 1:1000 |
| Rabbit monoclonal (D6H9) anti-phosphorylated ATM (Ser1981) | Cell Signaling | #5883 | 1:1000 |
| Rabbit monoclonal (133D3) anti-phosphorylated Chk1 (Ser345) | Cell Signaling | #2348 | 1:1000 |
| Rabbit monoclonal (E1S3S) anti-ATR | Cell Signaling | #13934 | 1:1000 |
| Rabbit monoclonal (D2E2) anti-ATM | Cell Signaling | #2873 | 1:1000 |
| Mouse monoclonal (2G1D5) anti-Chk1 | Cell Signaling | #2360 | 1:1000 |
| Goat Anti-Rabbit Immunoglobulins/HRP | Dako | P0448 | 1:5000 |
| Goat Anti-Mouse Immunoglobulins/HRP | Dako | P0447 | 1:5000 |

**Table S3. Primer sequences for qPCR.**

| Genes | Forward primer (5'-3') | Reverse primer (5'-3') |
|---|---|---|
| **For real-time qPCR** | | |
| MCM6 | GAGGAACTGATTCGTCCTGAGA | CAAGGCCCGACACAGGTAAG |
| YAP | TAGCCCTGCGTAGCCAGTTA | TCATGCTTAGTCCACTGTCTGT |
| β-actin | AGAGCTACGAGCTGCCTGAC | AGCACTGTGTTGGCGTACAG |
| **For ChIP-PCR** | | |
| MCM6 | TCACACTGTGTTGCCCAGG | AATTAGTCGGGCGTGGTGG |

**Table S4. Top 15 compounds identified from high-throughput virtual screening.**

| No. | Compounds | CAS No. | MW | Rotatable bonds | No. HBA | No. HBD | TPSA (Å$^2$) | Docking score |
|---|---|---|---|---|---|---|---|---|
| 1 | Parishin C | 174972-80-6 | 728.65 | 15 | 18 | 10 | 308.89 | -13.394 |
| 2 | Validamycin A | 37248-47-8 | 497.49 | 7 | 14 | 12 | 253.02 | -12.226 |
| 3 | Secoisolariciresinol diglucoside | 257930-74-8 | 686.70 | 15 | 16 | 10 | 257.68 | -12.131 |
| 4 | Isorhamnetin-3-O-neohespeidoside | 55033-90-4 | 624.54 | 7 | 16 | 9 | 258.43 | -11.918 |
| 5 | **Purpureaside C** | 108648-07-3 | 786.73 | 13 | 20 | 12 | 324.44 | -11.804 |
| 6 | Eriocitrin | 13463-28-0 | 596.53 | 6 | 15 | 9 | 245.29 | -11.724 |
| 7 | 1,4-b-D-Xylopentaose | 49694-20-4 | 678.59 | 12 | 21 | 12 | 333.67 | -11.688 |
| 8 | α,β-Methylene ATP trisodium | 1343364-54-4 | 505.21 | 11 | 16 | 4 | 236.9 | -11.668 |
| 9 | Ligustroflavone | 260413-62-5 | 724.66 | 8 | 18 | 10 | 287.89 | -11.606 |
| 10 | ATP | 56-65-5 | 507.18 | 8 | 14 | 7 | 279.13 | -11.589 |
| 11 | Uridine 5′-diphosphoglucose disodium salt | 28053-08-9 | 566.30 | 11 | 18 | 7 | 274.99 | -11.573 |
| 12 | Xylotetraose | 22416-58-6 | 546.47 | 10 | 17 | 10 | 274.75 | -11.545 |
| 13 | Uridine-5'-diphosphate disodium salt | 27821-45-0 | 404.16 | 6 | 12 | 4 | 223.5 | -11.539 |
| 14 | Rebaudioside A | 58543-16-1 | 967.01 | 12 | 23 | 14 | 374.13 | -11.363 |
| 15 | Oroxin B | 114482-86-9 | 594.52 | 7 | 15 | 9 | 249.2 | -11.346 |

MW: molecular weight; HBA: H-bond acceptors; HBD: H-bond donors; TPSA: topological polar surface area.

**Table S5. Clinicopathologic features of MCM6 protein expression in GC from Hong Kong tissue microarray cohort.**

| Variables | Low MCM6 (n = 149) | High MCM6 (n = 115) | *P* value |
|---|---|---|---|
| **Age, year, mean ± SD** | 63.1 ± 12.9 | 64.5 ± 12.5 | 0.353 |
| **Gender, n (%)** | | | 0.670 |
| Male | 96 (55.5) | 77 (44.5) | |
| Female | 53 (58.2) | 38 (41.8) | |
| **Lauren subtype, n (%)** | | | 0.997 |
| Diffuse | 70 (56.5) | 54 (43.5) | |
| Intestinal | 79 (56.4) | 61 (43.6) | |
| **Tumor grade, n (%)** | | | 0.938 |
| I | 5 (55.6) | 4 (44.4) | |
| II | 55 (57.9) | 40 (42.1) | |
| III | 89 (55.6) | 71 (44.4) | |
| **TNM stage, n (%)** | | | 0.684 |
| I | 32 (55.2) | 26 (44.8) | |
| II | 20 (62.5) | 12 (37.5) | |
| III | 50 (59.5) | 34 (40.5) | |
| IV | 47 (52.2) | 43 (47.8) | |
| **Lymph node metastasis, n (%)** | | | 0.423 |
| Positive | 113 (55.1) | 92 (44.9) | |
| Negative | 36 (61.0) | 23 (39.0) | |

TNM, tumor node metastasis.

**Table S6. Cox regression analysis of potential poor prognostic factors for GC patients from Hong Kong cohort.**

| Variables | Univariate Cox regression analysis | | Multivariate Cox regression analysis | |
|---|---|---|---|---|
| | HR (95% CI) | *P* value | HR (95% CI) | *P* value |
| **Age (years)** | | | | |
| >60 vs. ≤60 | 1.433 (1.001-2.050) | 0.049 | 1.837 (1.279-2.638) | 0.001 |
| **Gender** | | | | |
| Male vs. Female | 0.821 (0.580-1.163) | 0.266 | --- | --- |
| **Lauren subtype** | | | | |
| Diffuse vs. Intestinal | 1.904 (1.353-2.679) | 0.000 | 1.479 (0.891-2.452) | 0.130 |
| **Tumor grade** | | | | |
| III vs. I and II | 1.277 (1.067-1.527) | 0.008 | 0.956 (0.734-1.245) | 0.739 |
| **TNM stage** | | | | |
| III and IV vs. I and II | 2.434 (1.911-3.098) | 0.000 | 2.024 (1.435-2.855) | 0.000 |
| **Lymph node metastasis** | | | | |
| Positive vs. Negative | 5.292 (2.914-9.612) | 0.000 | 1.721 (0.752-3.943) | 0.199 |
| **MCM6 protein expression** | | | | |
| High vs. Low | 1.577 (1.125-2.212) | 0.008 | 1.545 (1.093-2.184) | 0.014 |

CI, confidence interval; HR, hazard ratio; TNM, tumor node metastasis.

**Table S7. List of proteins have physical interactions with MCM6.**

| Interactor A | Interactor B | Experimental System | Type | Throughput | Scores | Publications (PMID) |
|---|---|---|---|---|---|---|
| MKI67 | MCM6 | Affinity Capture-MS | physical | High Throughput | 68.7 | 26949251 |
| CCNF | MCM6 | Affinity Capture-MS | physical | High Throughput | 6.119 | 33729478 |
| FGD2 | MCM6 | Affinity Capture-MS | physical | High Throughput | 4.790311538 | 32203420 |
| DEPDC1B | MCM6 | Affinity Capture-MS | physical | High Throughput | 3.114018714 | 32203420 |
| USP7 | MCM6 | Affinity Capture-MS | physical | High Throughput | 2.49 | 19615732 |
| TNFAIP3 | MCM6 | Affinity Capture-MS | physical | High Throughput | 1.04 | 19615732 |
| USP49 | MCM6 | Affinity Capture-MS | physical | High Throughput | 1.04 | 19615732 |
| MCM6 | MCM3 | Co-fractionation | physical | High Throughput | 1 | 26344197 |
| MCM6 | MCM5 | Co-fractionation | physical | High Throughput | 1 | 26344197 |
| MCM2 | MCM6 | Co-fractionation | physical | High Throughput | 1 | 26344197 |
| MCM4 | MCM6 | Co-fractionation | physical | High Throughput | 1 | 26344197 |
| MCM6 | MCM7 | Co-fractionation | physical | High Throughput | 1 | 26344197 |
| MCM6 | MCMBP | Co-fractionation | physical | High Throughput | 1 | 26344197 |
| MCM2 | MCM6 | Affinity Capture-MS | physical | High Throughput | 0.99986232 | 33961781 |

| | | | | | | |
|---|---|---|---|---|---|---|
| ASF1A | MCM6 | Affinity Capture-MS | physical | High Throughput | 0.999188517 | 28514442 |
| MCM2 | MCM6 | Co-fractionation | physical | High Throughput | 0.998 | 22939629 |
| ASF1A | MCM6 | Affinity Capture-MS | physical | High Throughput | 0.996927062 | 33961781 |
| H2AFX | MCM6 | Affinity Capture-MS | physical | High Throughput | 0.996572984 | 33961781 |
| ASF1B | MCM6 | Affinity Capture-MS | physical | High Throughput | 0.9954816 | 28514442 |
| MCM4 | MCM6 | Co-fractionation | physical | High Throughput | 0.994 | 22939629 |
| HIST1H2BA | MCM6 | Affinity Capture-MS | physical | High Throughput | 0.989445436 | 26186194 |
| HIST1H3H | MCM6 | Affinity Capture-MS | physical | High Throughput | 0.987932919 | 33961781 |
| HIST1H4B | MCM6 | Affinity Capture-MS | physical | High Throughput | 0.983718826 | 33961781 |
| ASF1B | MCM6 | Affinity Capture-MS | physical | High Throughput | 0.982780246 | 33961781 |
| HIST1H3A | MCM6 | Affinity Capture-MS | physical | High Throughput | 0.97702389 | 28514442 |
| MCM6 | MCM7 | Co-fractionation | physical | High Throughput | 0.973 | 22939629 |
| HIST1H2BA | MCM6 | Affinity Capture-MS | physical | High Throughput | 0.967116608 | 28514442 |
| HIST1H4I | MCM6 | Affinity Capture-MS | physical | High Throughput | 0.962819678 | 33961781 |
| H3F3B | MCM6 | Affinity Capture-MS | physical | High Throughput | 0.954486055 | 33961781 |
| HIST1H2AE | MCM6 | Affinity Capture-MS | physical | High Throughput | 0.946957461 | 33961781 |

| | | | | | | |
|---|---|---|---|---|---|---|
| MCM3 | MCM6 | Affinity Capture-MS | physical | High Throughput | 0.944561808 | 28514442 |
| H2AFX | MCM6 | Affinity Capture-MS | physical | High Throughput | 0.926195261 | 28514442 |
| HIST1H4F | MCM6 | Affinity Capture-MS | physical | High Throughput | 0.920560389 | 33961781 |
| HIST1H4A | MCM6 | Affinity Capture-MS | physical | High Throughput | 0.909759502 | 33961781 |
| MCM10 | MCM6 | Affinity Capture-MS | physical | High Throughput | 0.907069568 | 26186194 |
| HIST1H2AB | MCM6 | Affinity Capture-MS | physical | High Throughput | 0.899304838 | 33961781 |
| HIST1H3F | MCM6 | Affinity Capture-MS | physical | High Throughput | 0.899262594 | 33961781 |
| HIST1H4C | MCM6 | Affinity Capture-MS | physical | High Throughput | 0.898491564 | 33961781 |
| HIST1H4L | MCM6 | Affinity Capture-MS | physical | High Throughput | 0.893576767 | 33961781 |
| HIST1H3A | MCM6 | Affinity Capture-MS | physical | High Throughput | 0.886145524 | 33961781 |
| SSRP1 | MCM6 | Affinity Capture-MS | physical | High Throughput | 0.881012864 | 28514442 |
| HIST1H2BA | MCM6 | Affinity Capture-MS | physical | High Throughput | 0.878743859 | 33961781 |
| SPIN2B | MCM6 | Affinity Capture-MS | physical | High Throughput | 0.873822839 | 26186194 |
| MCM3 | MCM6 | Affinity Capture-MS | physical | High Throughput | 0.872802882 | 33961781 |
| HIST1H4A | MCM6 | Affinity Capture-MS | physical | High Throughput | 0.870251555 | 28514442 |
| HIST1H3B | MCM6 | Affinity Capture-MS | physical | High Throughput | 0.851695182 | 33961781 |

| | | | | | | |
|---|---|---|---|---|---|---|
| MCM3 | MCM6 | Co-fractionation | physical | High Throughput | 0.843 | 22939629 |
| MCM5 | MCM6 | Co-fractionation | physical | High Throughput | 0.838 | 22939629 |
| MCM10 | MCM6 | Affinity Capture-MS | physical | High Throughput | 0.822595333 | 28514442 |
| MCM6 | SF3B1 | Co-fractionation | physical | High Throughput | 0.821 | 22939629 |
| ING5 | MCM6 | Affinity Capture-MS | physical | High Throughput | 0.807339349 | 33961781 |
| MCM6 | SF3A1 | Co-fractionation | physical | High Throughput | 0.779 | 22939629 |
| SPIN2B | MCM6 | Affinity Capture-MS | physical | High Throughput | 0.765446546 | 28514442 |
| DCTN2 | MCM6 | Co-fractionation | physical | High Throughput | 0.764 | 22939629 |
| TAF10 | MCM6 | Affinity Capture-MS | physical | High Throughput | 0.677452 | 17643375 |
| MAPRE2 | MCM6 | Co-fractionation | physical | High Throughput | 0.087790617 | 26344197 |
| SDHA | MCM6 | Affinity Capture-MS | physical | High Throughput | 0.000446535 | 29128334 |
| SOD1 | MCM6 | Affinity Capture-MS | physical | High Throughput | 0.000296463 | 29128334 |
| DLD | MCM6 | Affinity Capture-MS | physical | High Throughput | 0.000241786 | 29128334 |

**Table S8. siRNA used in this study.**

| siRNA | Sequence (5'-3') | |
|---|---|---|
| siMCM6-1 | Sense | GGAACAAUUUAACCAGCAATT |
| | Antisense | UUGCUGGUUAAAUUGUUCCTT |
| siMCM6-2 | Sense | GCUCCCAAAGCCUCCUUAATT |
| | Antisense | UUAAGGAGGCUUUGGGAGCTT |

| siRNA | Source | Cat. No. |
|---|---|---|
| siYAP-1 | QIAGEN | SI02662954 |
| siYAP-2 | QIAGEN | SI04438651 |
| Negative control siRNA | QIAGEN | 1027281 |

**Table S9.** **shRNA and DNA plasmids.**

| **shRNA knockdown** | | |
|---|---|---|
| **shRNA** | **Target sequence (5'-3')** | |
| shMCM6-1 | TACAGCTAAGAGCCAATTTCTCAAGCACG | |
| shMCM6-2 | GCATTCCAAGACCTGCCTACCAGACACAA | |
| **Overexpression** | | |
| **Gene** | **Genbank No.** | **Source** |
| Empty vector (EV) | N/A | GeneChem |
| MCM6 | NM_005915 | GeneChem/31108-15 |

**Table S10. Antibodies used for immunohistochemistry.**

| Antibodies | Source | Cat. No. | Working concentration | Antigen retrieval buffer |
|---|---|---|---|---|
| Rabbit monoclonal anti-MCM6 | Abcam | ab201683 | 1:100 | Tris-EDTA |
| Rabbit monoclonal anti-YAP | Abcam | ab52771 | 1:100 | Tris-EDTA |
| Mouse monoclonal anti-PCNA | Immunoway | YM3031 | 1:200 | Tris-EDTA |
| Rabbit anti-phosphorylated AKT (Ser473) | Cell Signaling | #9271 | 1:100 | Tris-EDTA |
| Rabbit anti-Cleaved PARP | Cell Signaling | #9541 | 1:100 | Tris-EDTA |

**References**

1. Brunet A, Bonni A, Zigmond MJ, Lin MZ, Juo P, Hu LS, et al. Akt promotes cell survival by phosphorylating and inhibiting a Forkhead transcription factor. Cell. 1999; 96: 857-68.
